# Supplementary material for: Inherent fast inactivation particle of Nav channels as a new binding site for a neurotoxin
Source: EMBO J. 2025 Apr 22;44(11):3180–209. doi: 10.1038/s44318-025-00438-9 (PMC12130229; doi:10.1038/s44318-025-00438-9)
Supplement: Supplementary file 1 — Appendix [file 44318_2025_438_MOESM1_ESM.pdf]

Appendix Supplementary Information

N-terminal Edman degradation sequencing of rpTx1 (Cycle 1- Cycle 20)	Page 2- page 7
3’RACE data	Page 8
Electrospray ionization mass spectrum of recombinant WT-rpTx1 and rpTx1 mutants	Page 9-11

N-terminal Edman degradation sequencing:

Cycle 1

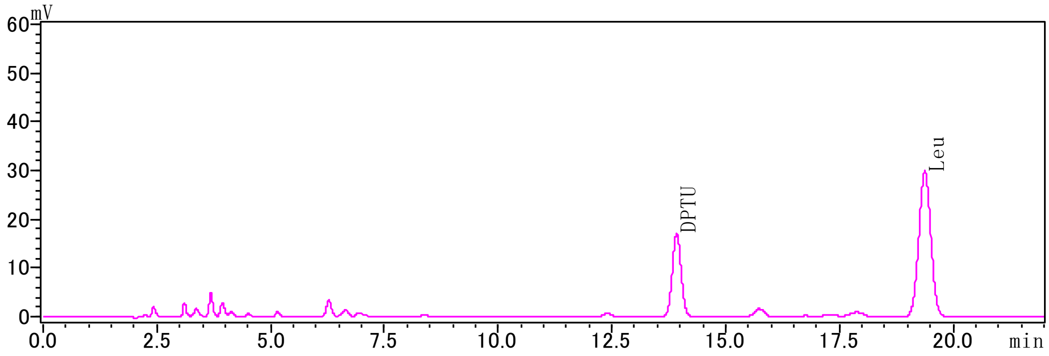

Cycle 2

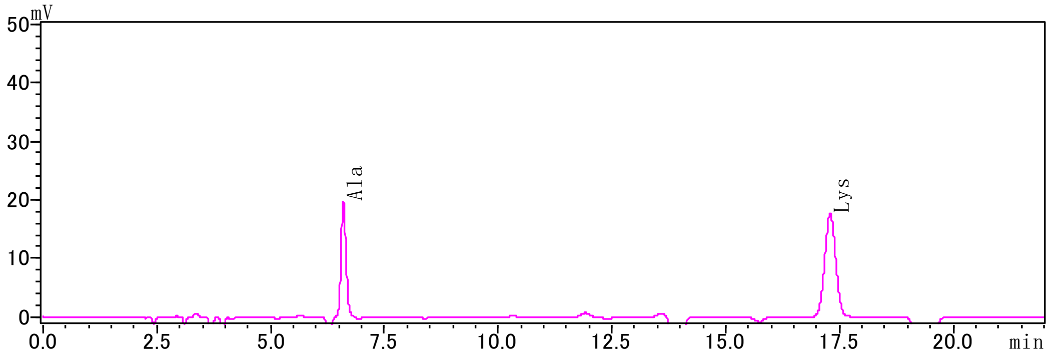

Cycle 3

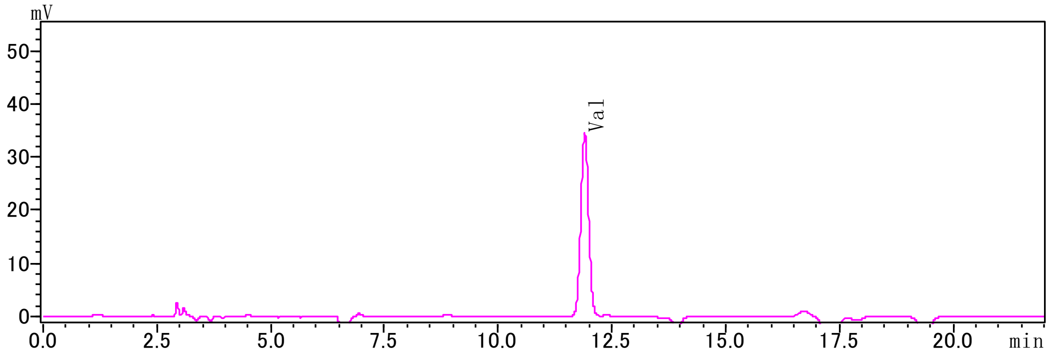

Cycle 4

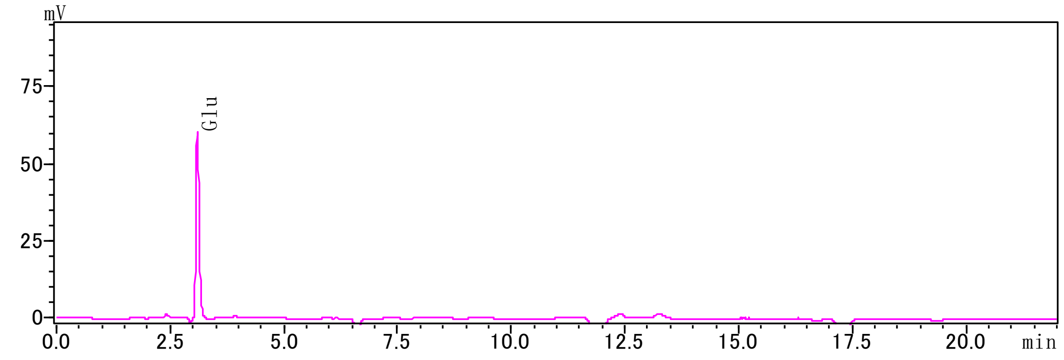

Cycle 5

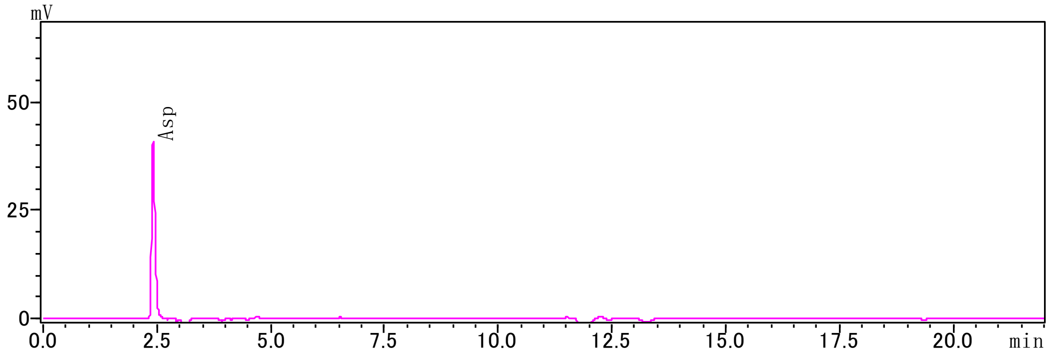

Cycle 6

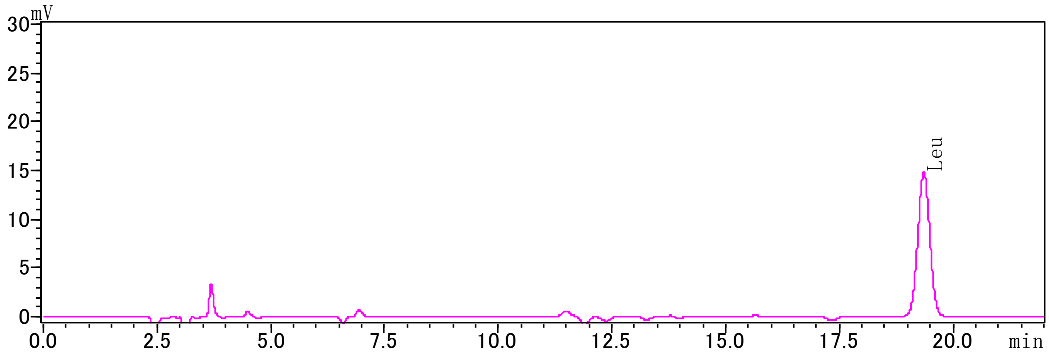

Cycle 7

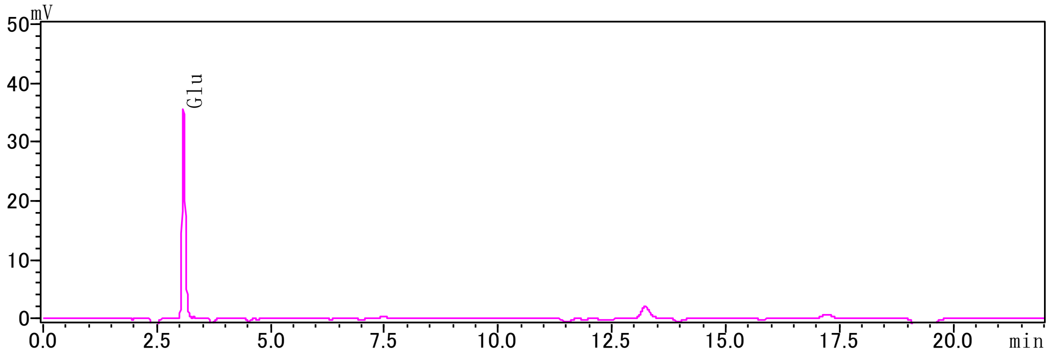

Cycle 8

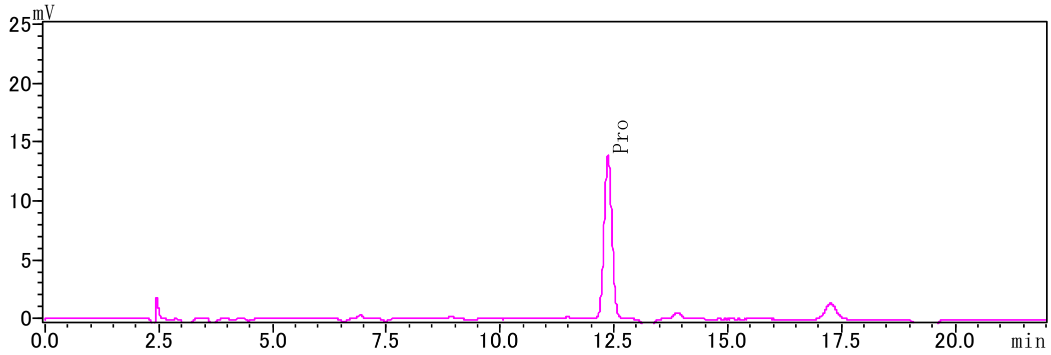

Cycle 9

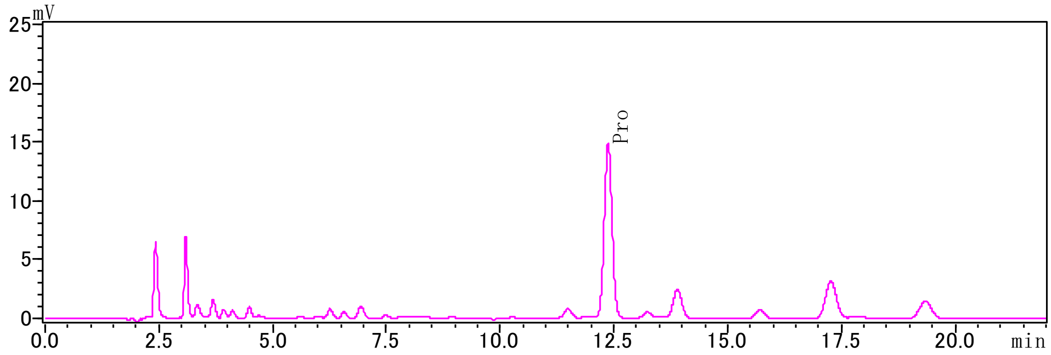

Cycle 10

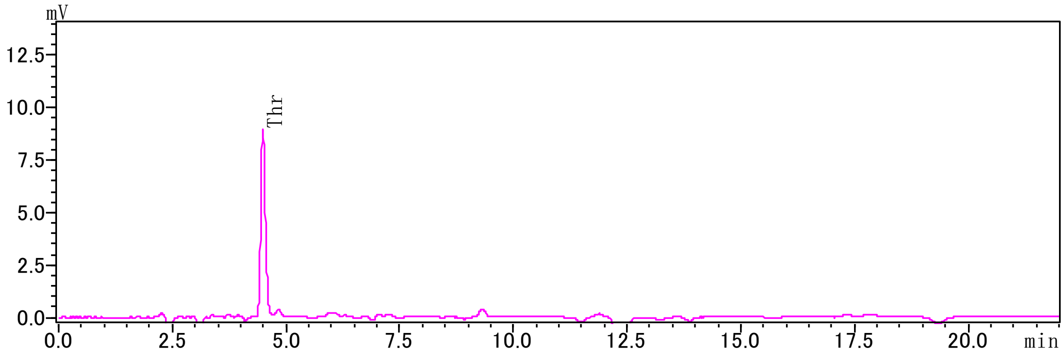

Cycle 11

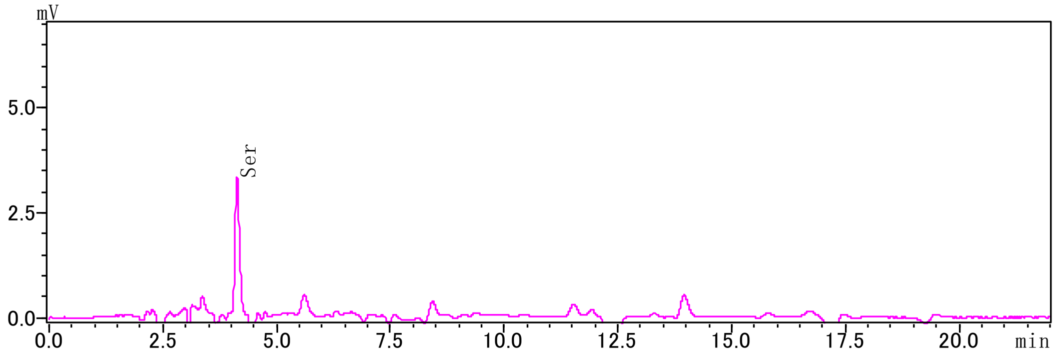

Cycle 12

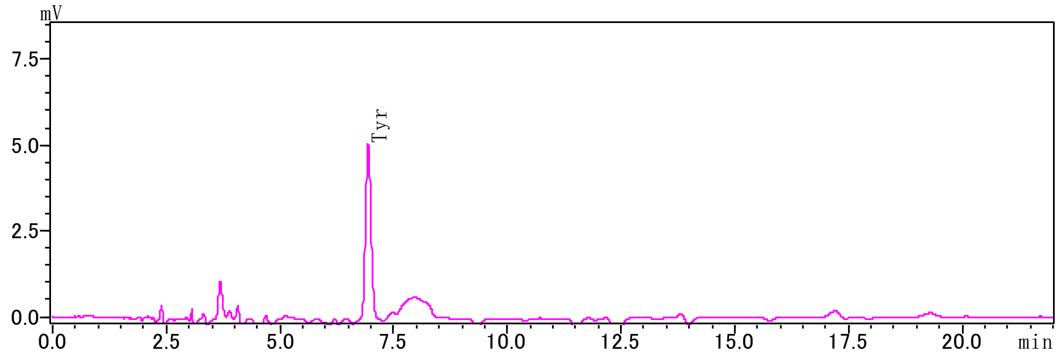

Cycle 13

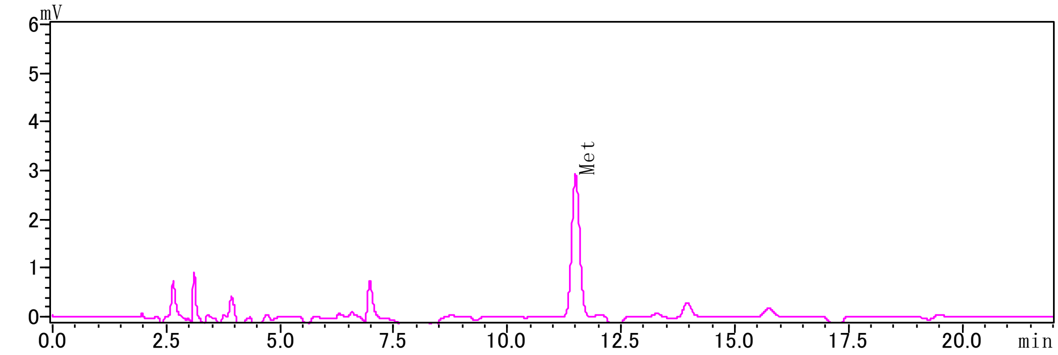

Cycle 14

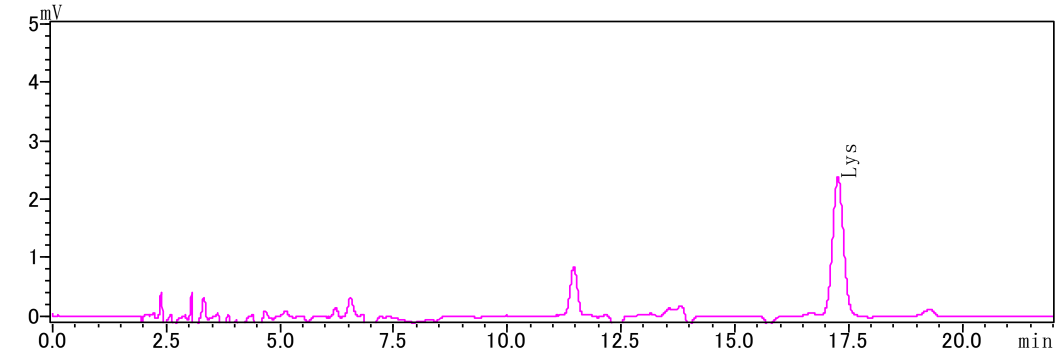

Cycle 15

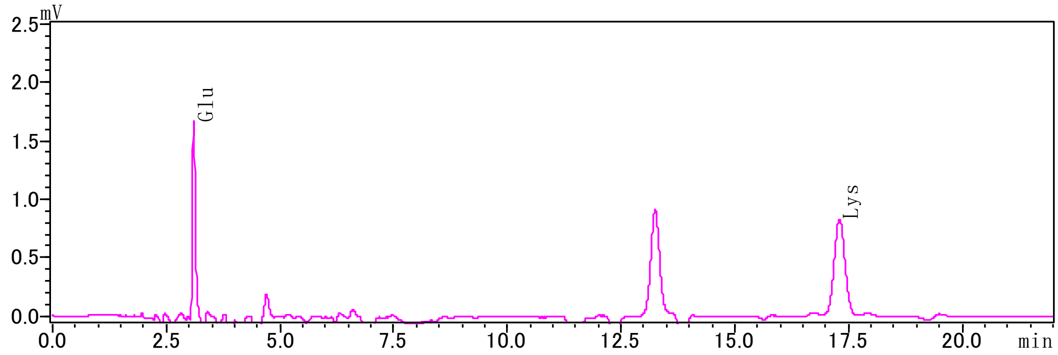

Cycle 16

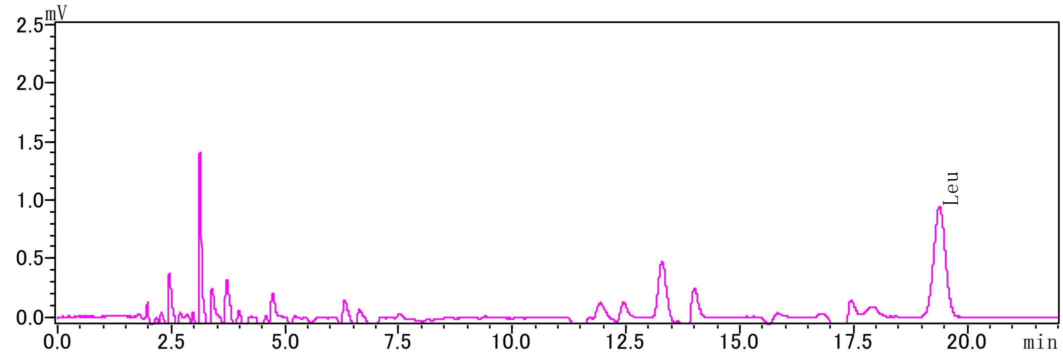

Cycle 17

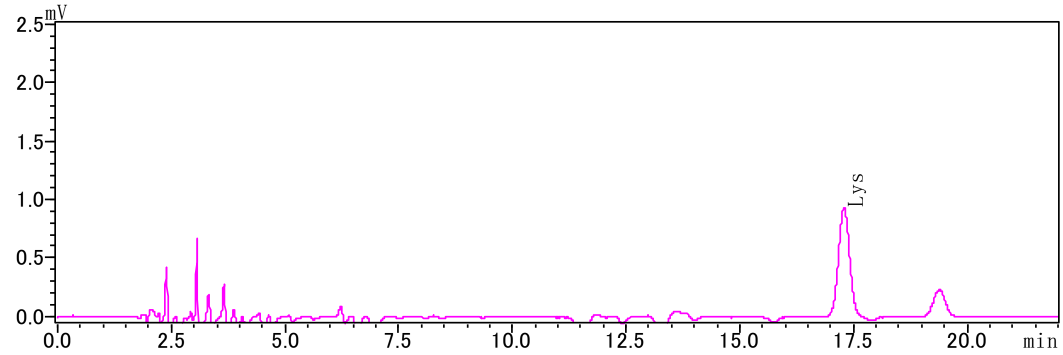

Cycle 18

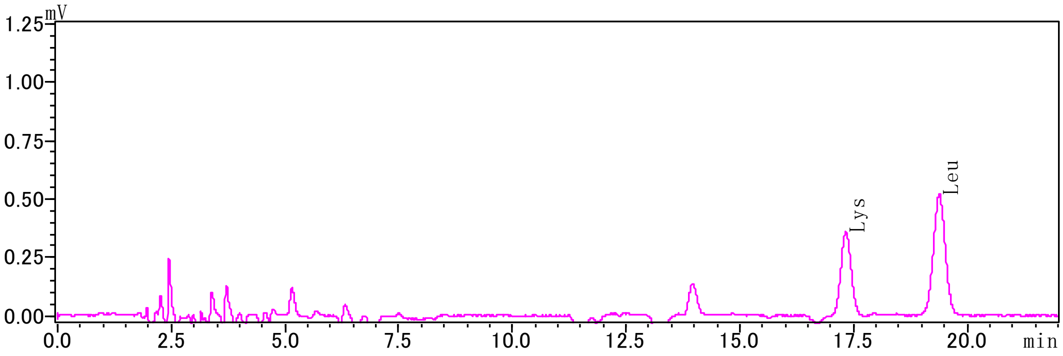

Cycle 19

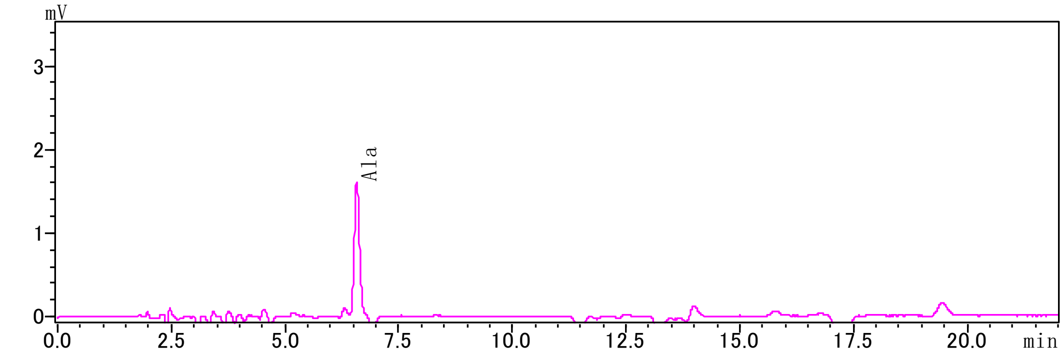

Cycle 20

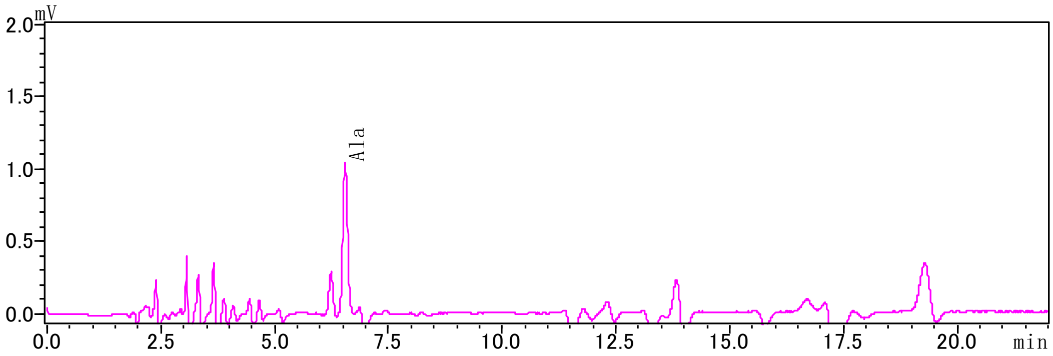

3’RACE data:

GTAGAAGATTTGGAGCCTCCGACATCCTACATGAAGGAACTGAAACTGGCT
V E D L E P P T S Y M K E L K L A
TTGGATAAAGATAAAAAAGATCCTAAACTGCAAGCTTATATTAAAAAAATC
L D K D K K D P K L Q A Y I K K I
TTTCCAGAGAACAGGAAAAGCTGTATGACAAACTGCAAACACGTTGAAGGA
F P E N R K S C M T N C K H V E G
TGTTTTTTGCTTTCTCCCGAATGCTGTCCAAAAATGACTTCAACTTGCCTT
C F L L S P E C C P K M T S T C L
GAGCTCGACATTGTGAAAGAACATATGAAAAAAACCAAAGGATAATTTACA
E L D I V K E H M K K T K G *
CAGGAACTAAATTATTTTAATATATTTTTTTTTTTTAAAAAAA

Electrospray ionization mass spectrum of recombinant WT-rpTx1 and rpTx1 mutants

WT

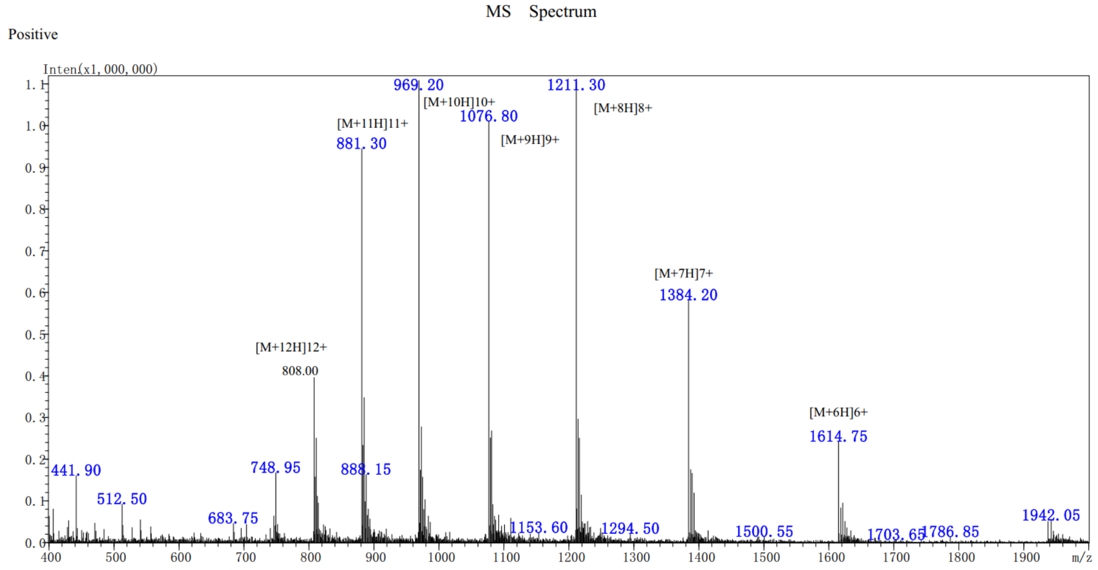

M45A

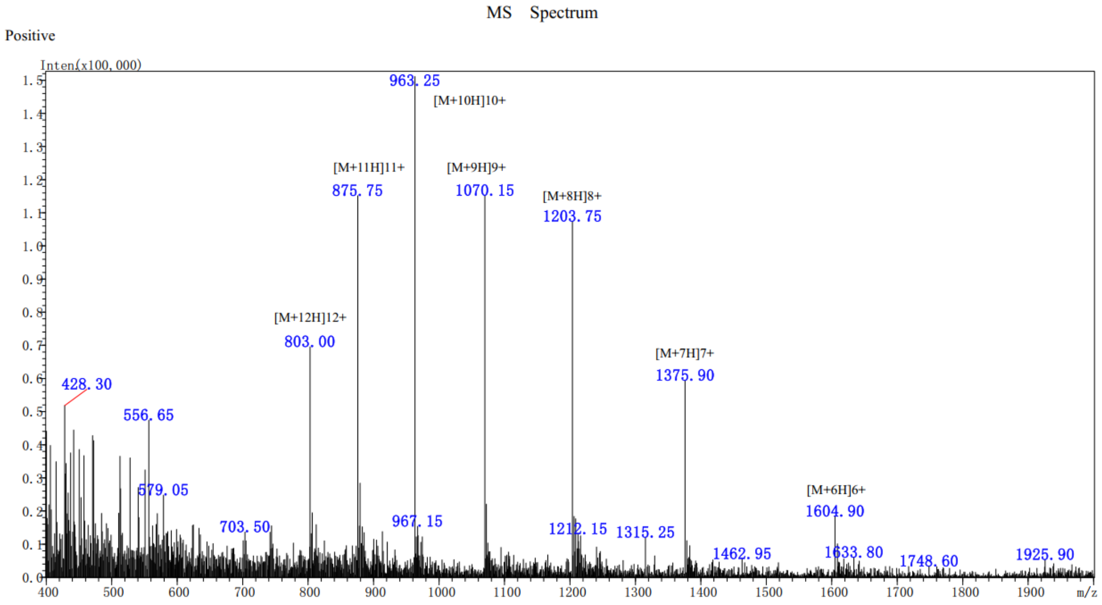

E60A

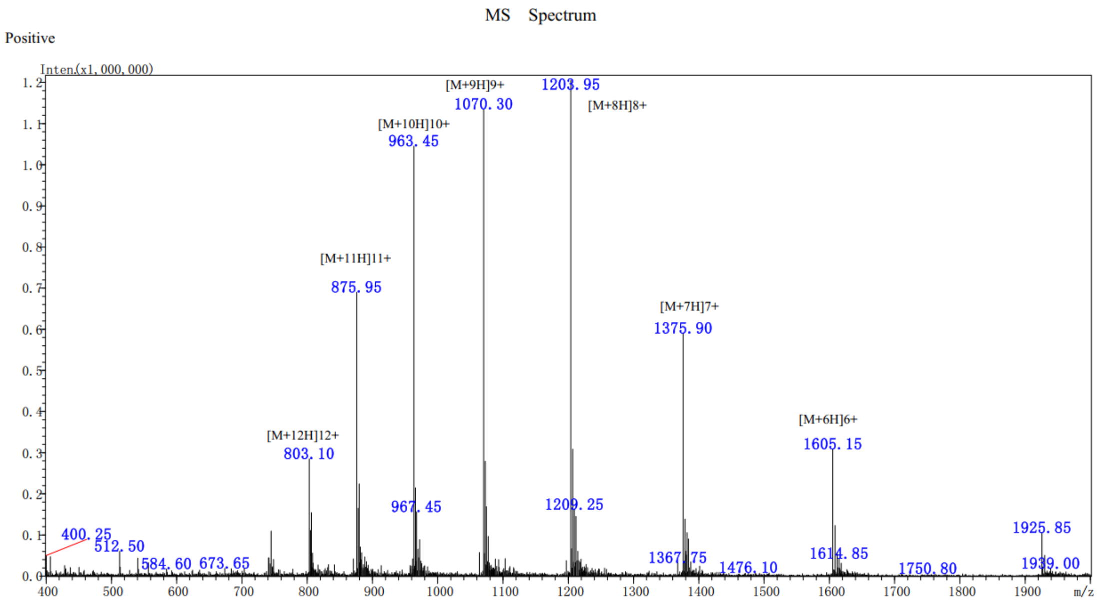
MS Spectrum
Positive
Inten.(x1,000,000)
[M+9H]9+
1070.30
1203.95
[M+8H]8+
[M+10H]10+
963.45
[M+11H]11+
875.95
[M+7H]7+
1375.90
[M+6H]6+
1605.15
[M+12H]12+
803.10
967.45
1209.25
400.25
512.50
584.60
673.65
1367.75
1476.10
1614.85
1750.80
1925.85
1939.00
m/z

T66A

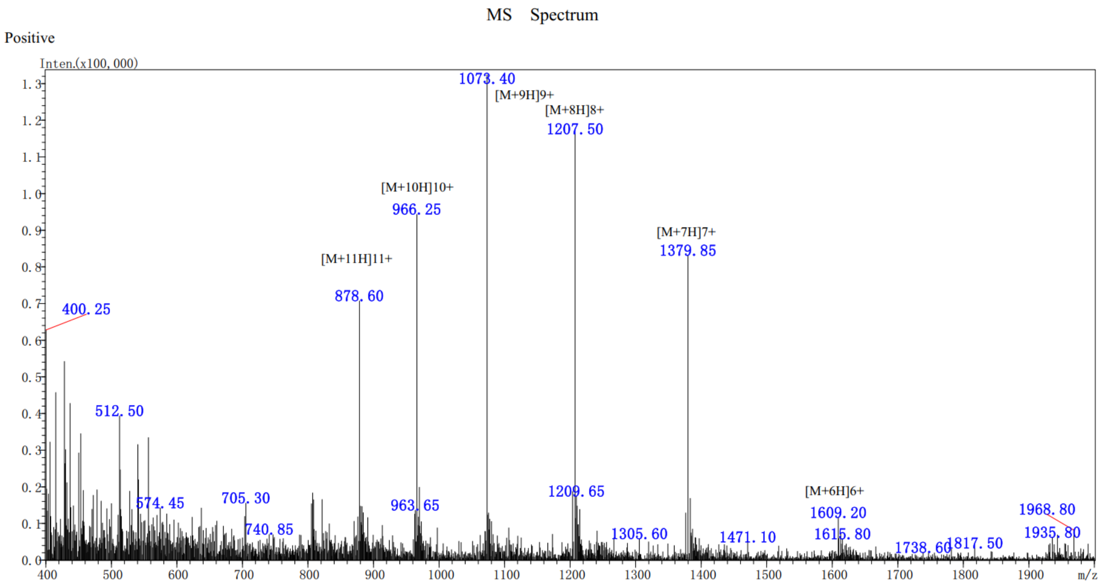
MS Spectrum
Positive
Inten.(x100,000)
1073.40
[M+9H]9+
[M+8H]8+
1207.50
[M+10H]10+
966.25
[M+7H]7+
1379.85
[M+11H]11+
878.60
400.25
512.50
574.45
705.30
740.85
963.65
1209.65
1305.60
1471.10
[M+6H]6+
1609.20
1615.80
1738.60
1817.50
1968.80
1935.80
m/z

rpTx1-8K/A

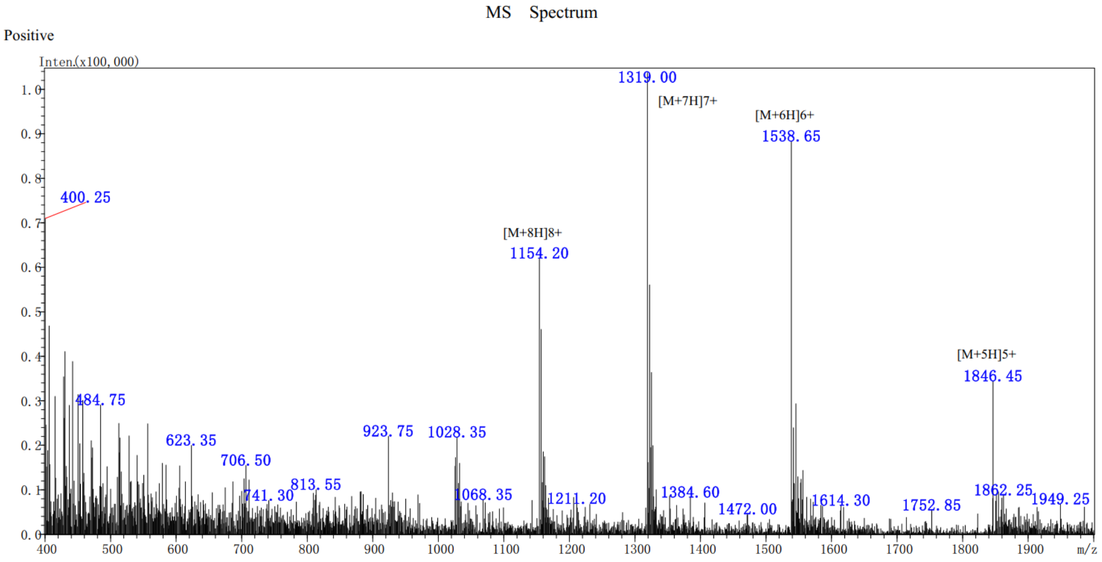
MS Spectrum
Positive
Inten.(x100,000)
1319.00
[M+7H]7+
[M+6H]6+
1538.65
400.25
[M+8H]8+
1154.20
[M+5H]5+
1846.45
484.75
623.35
923.75
1028.35
706.50
741.30
813.55
1068.35
1211.20
1384.60
1472.00
1614.30
1752.85
1862.25
1949.25
m/z
