# Supplementary figures and images for: Inherent fast inactivation particle of Nav channels as a new binding site for a neurotoxin
Source: EMBO J. 2025 Apr 22;44(11):3180–209. doi: 10.1038/s44318-025-00438-9 (PMC12130229; doi:10.1038/s44318-025-00438-9)

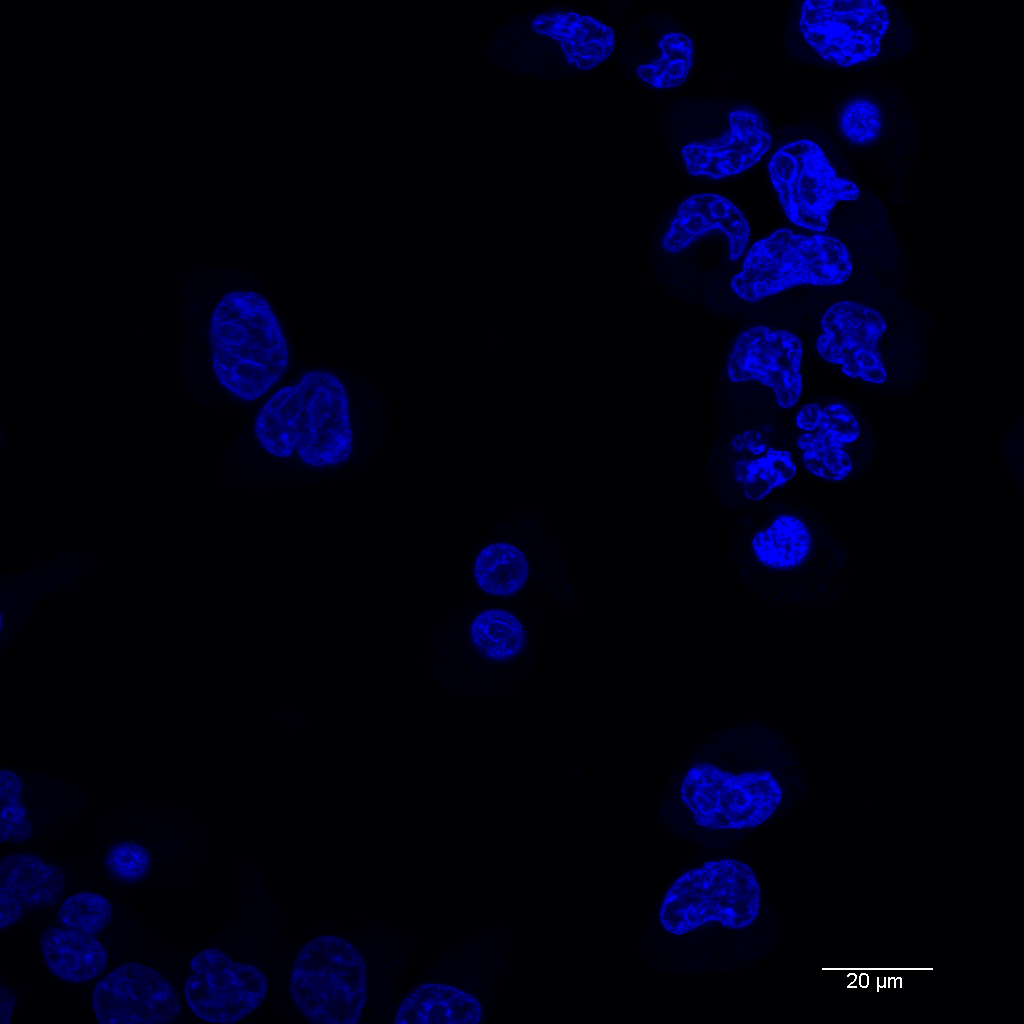

Supplement: Supplementary file 6 — Source data Fig. 2 [file 44318_2025_438_MOESM6_ESM.zip › Figure 2/Figure 2A/rpTx1 DAPI.tif]

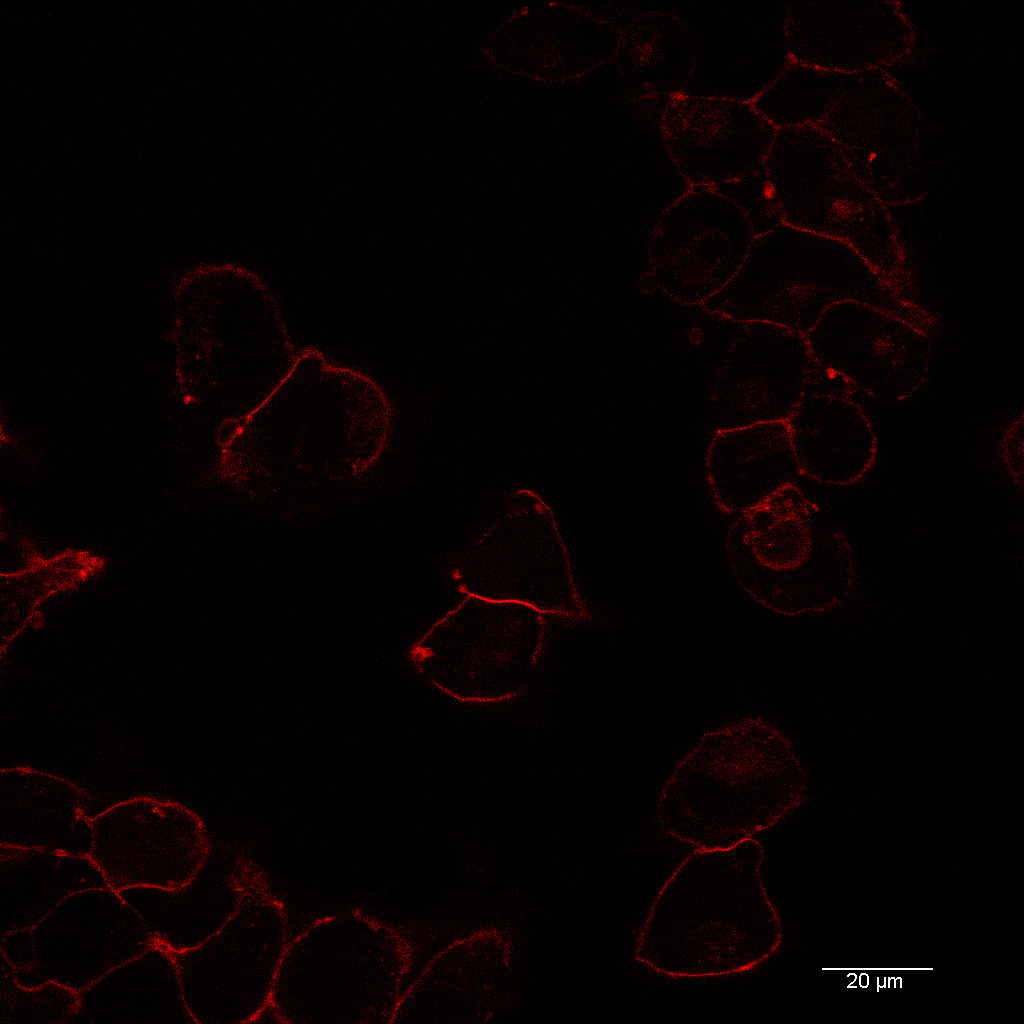

Supplement: Supplementary file 6 — Source data Fig. 2 [file 44318_2025_438_MOESM6_ESM.zip › Figure 2/Figure 2A/rpTx1 DiD.tif]

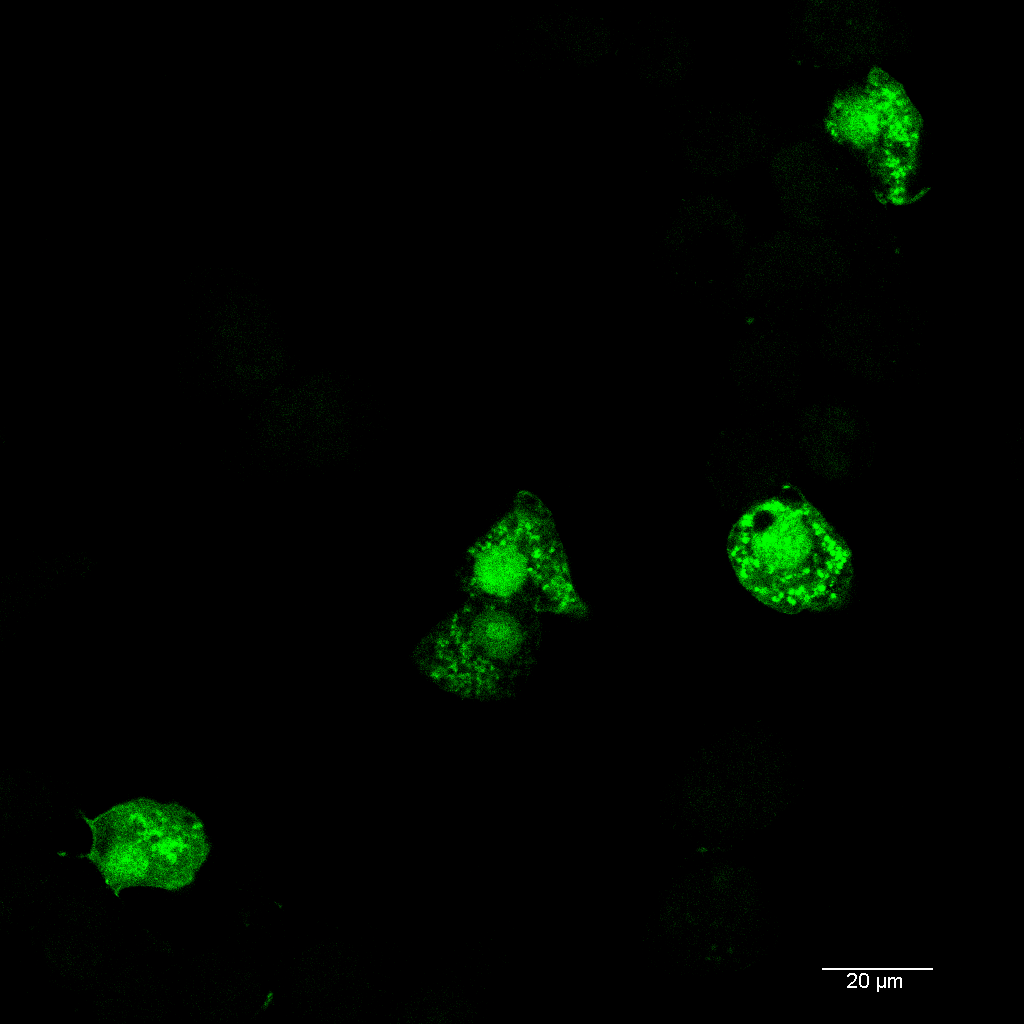

Supplement: Supplementary file 6 — Source data Fig. 2 [file 44318_2025_438_MOESM6_ESM.zip › Figure 2/Figure 2A/rpTx1 FITC.tif]

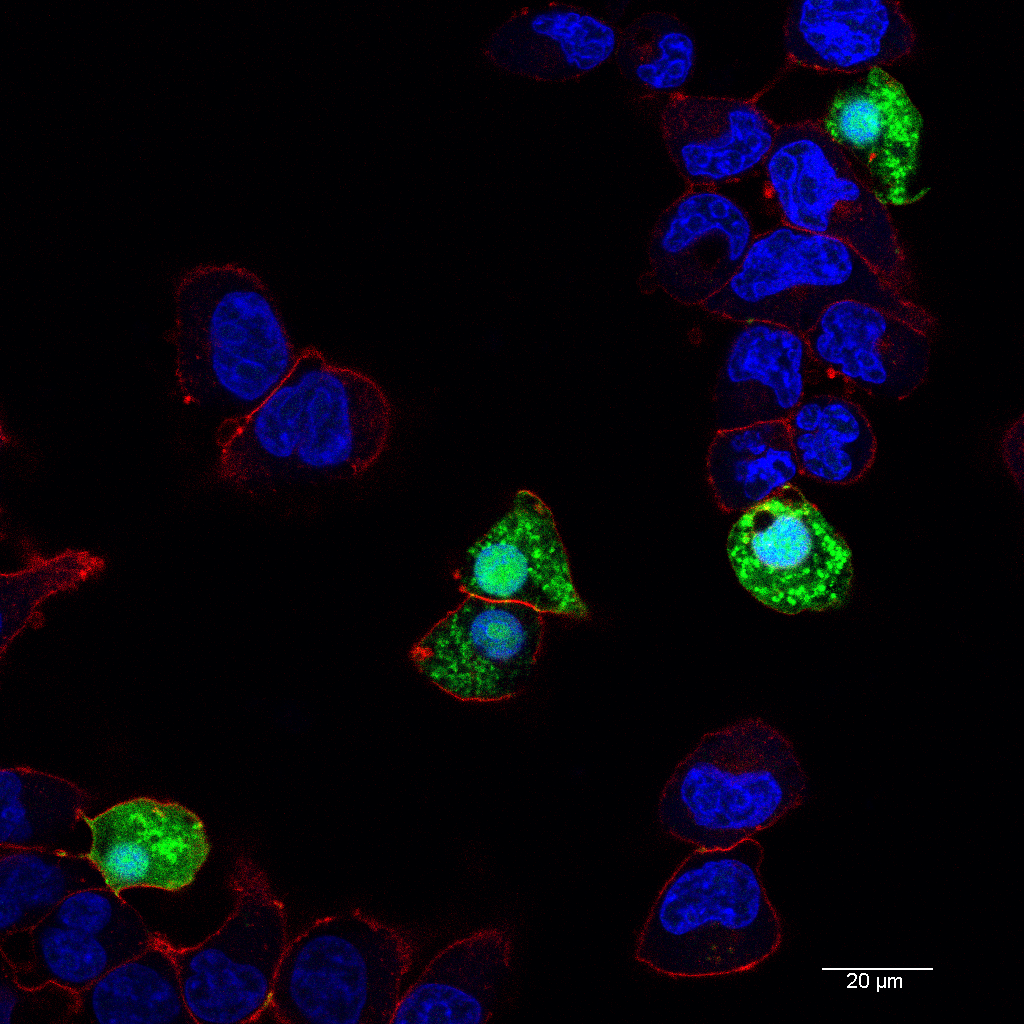

Supplement: Supplementary file 6 — Source data Fig. 2 [file 44318_2025_438_MOESM6_ESM.zip › Figure 2/Figure 2A/rpTx1 merge.tif]

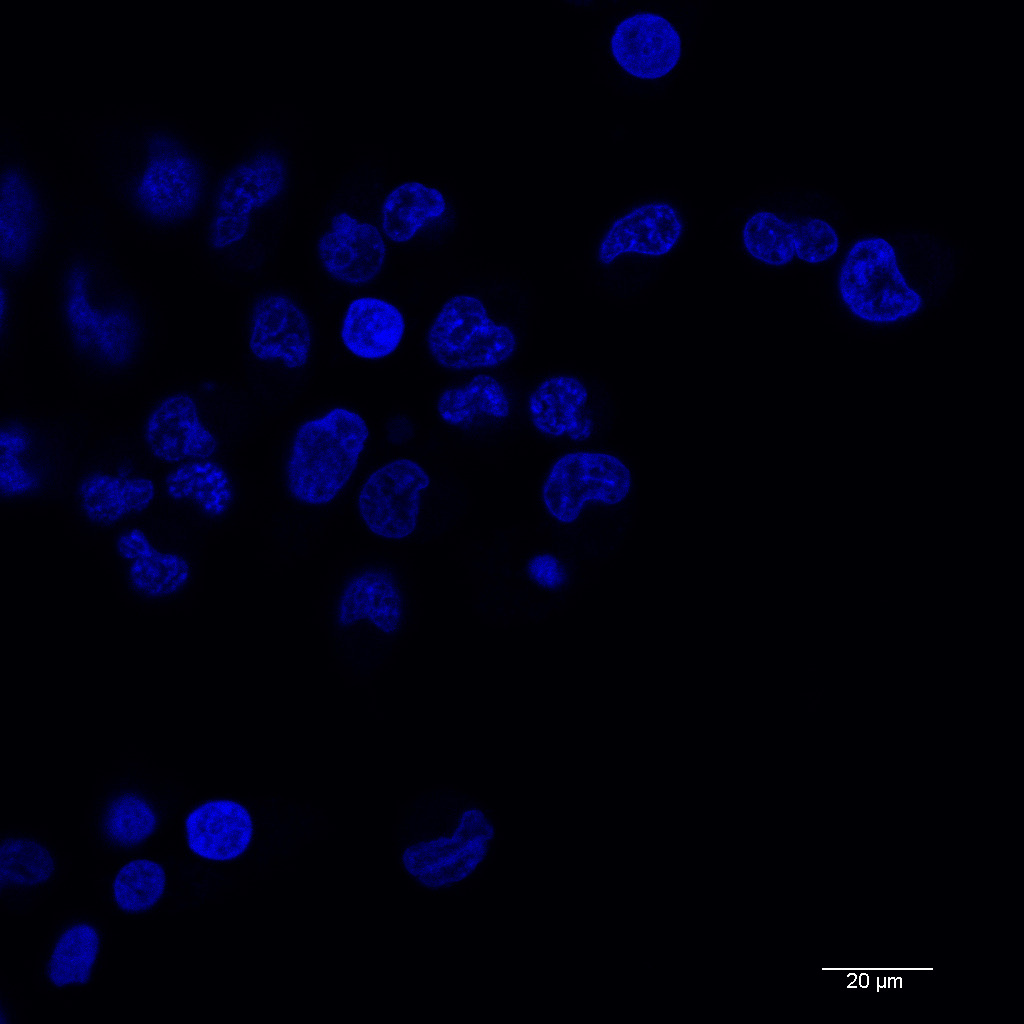

Supplement: Supplementary file 6 — Source data Fig. 2 [file 44318_2025_438_MOESM6_ESM.zip › Figure 2/Figure 2A/rpTx1-8KA DAPI.tif]

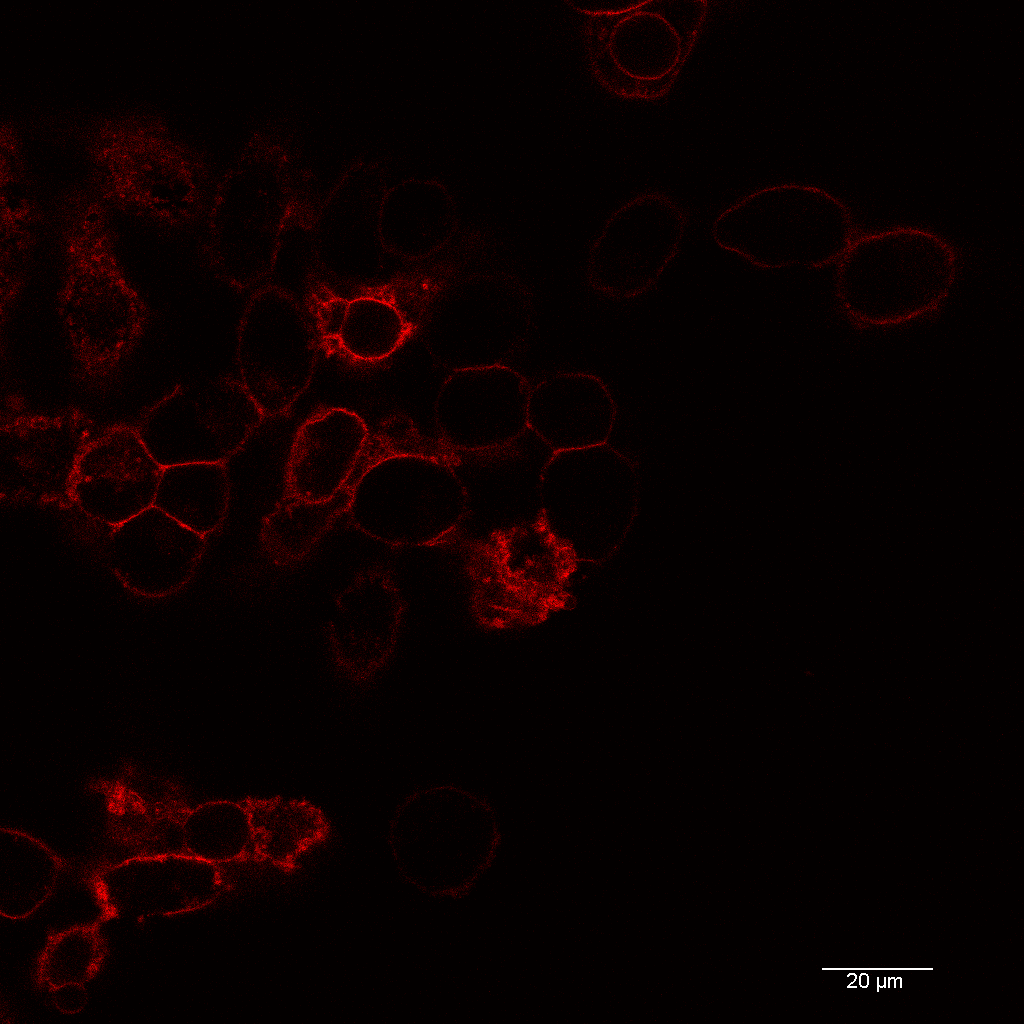

Supplement: Supplementary file 6 — Source data Fig. 2 [file 44318_2025_438_MOESM6_ESM.zip › Figure 2/Figure 2A/rpTx1-8KA DiD.tif]

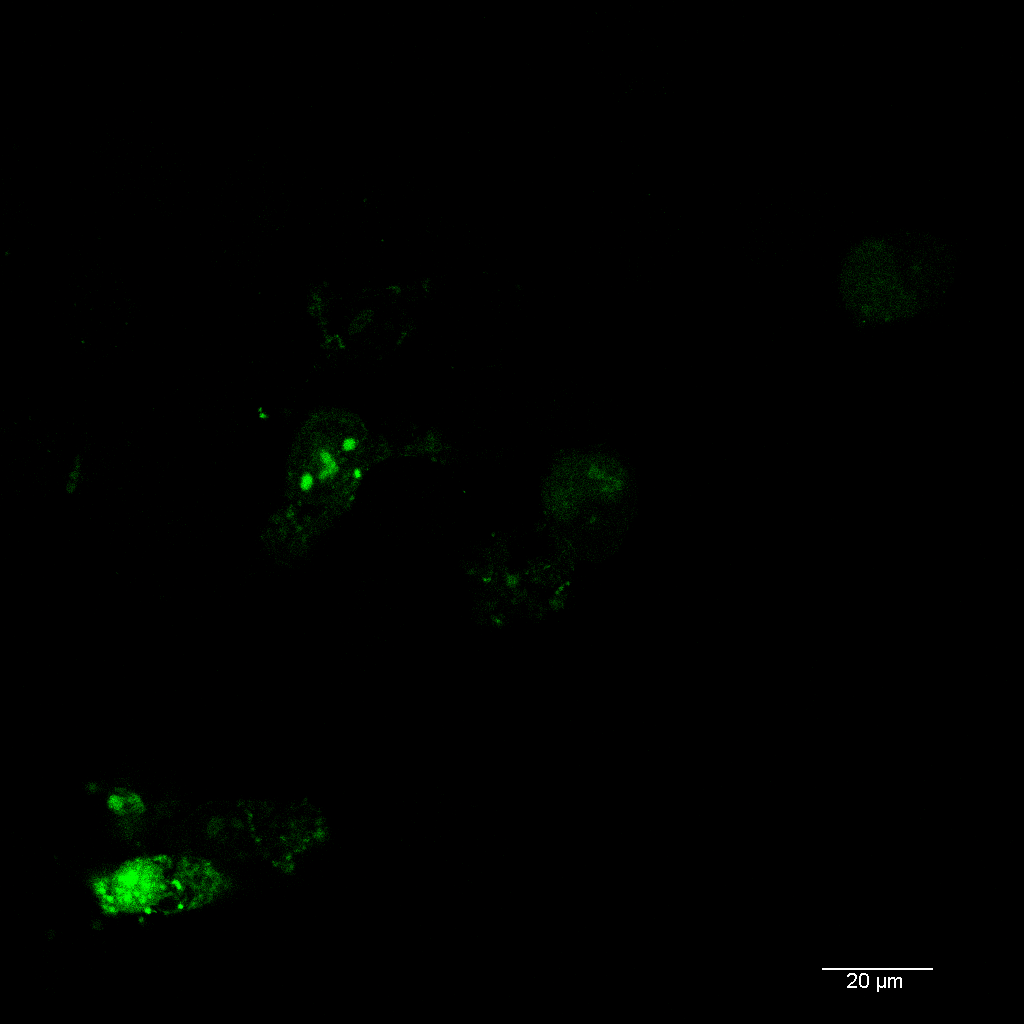

Supplement: Supplementary file 6 — Source data Fig. 2 [file 44318_2025_438_MOESM6_ESM.zip › Figure 2/Figure 2A/rpTx1-8KA FITC.tif]

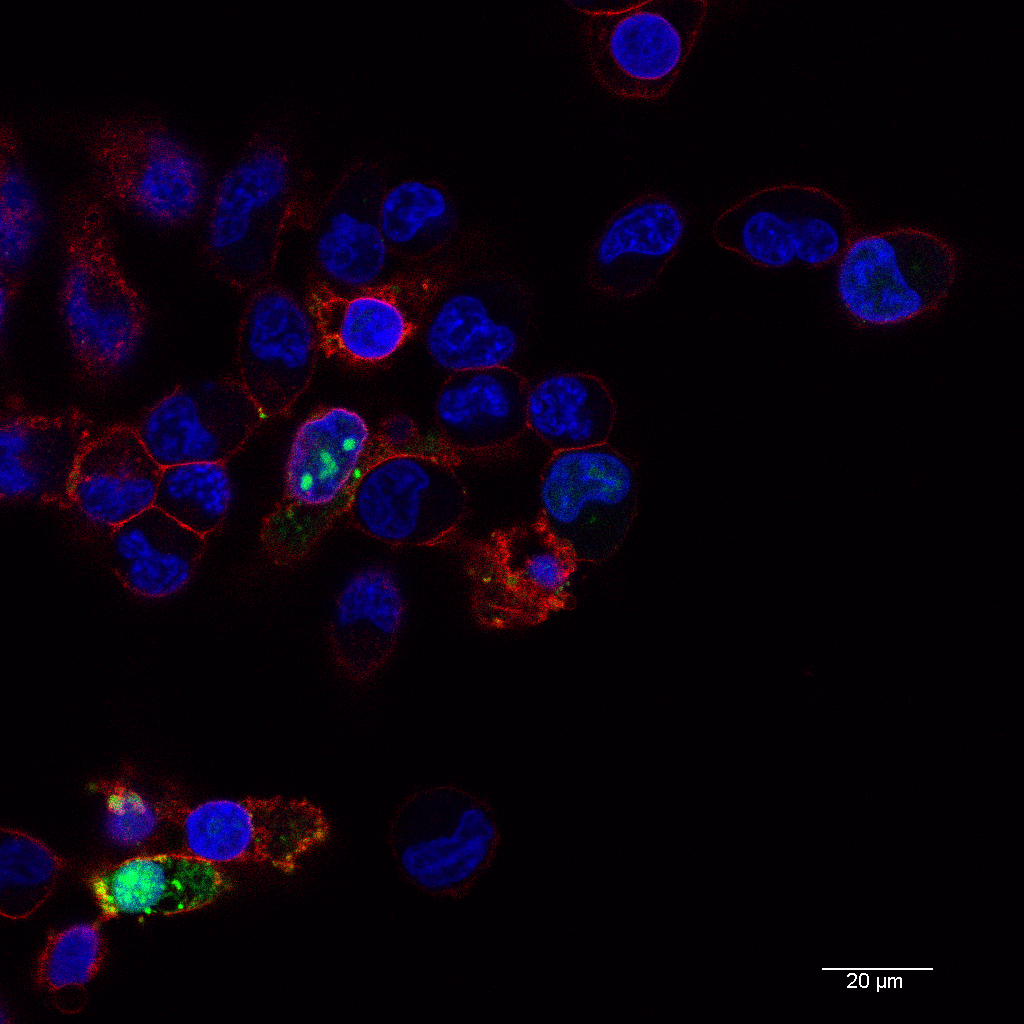

Supplement: Supplementary file 6 — Source data Fig. 2 [file 44318_2025_438_MOESM6_ESM.zip › Figure 2/Figure 2A/rpTx1-8KA merge.tif]

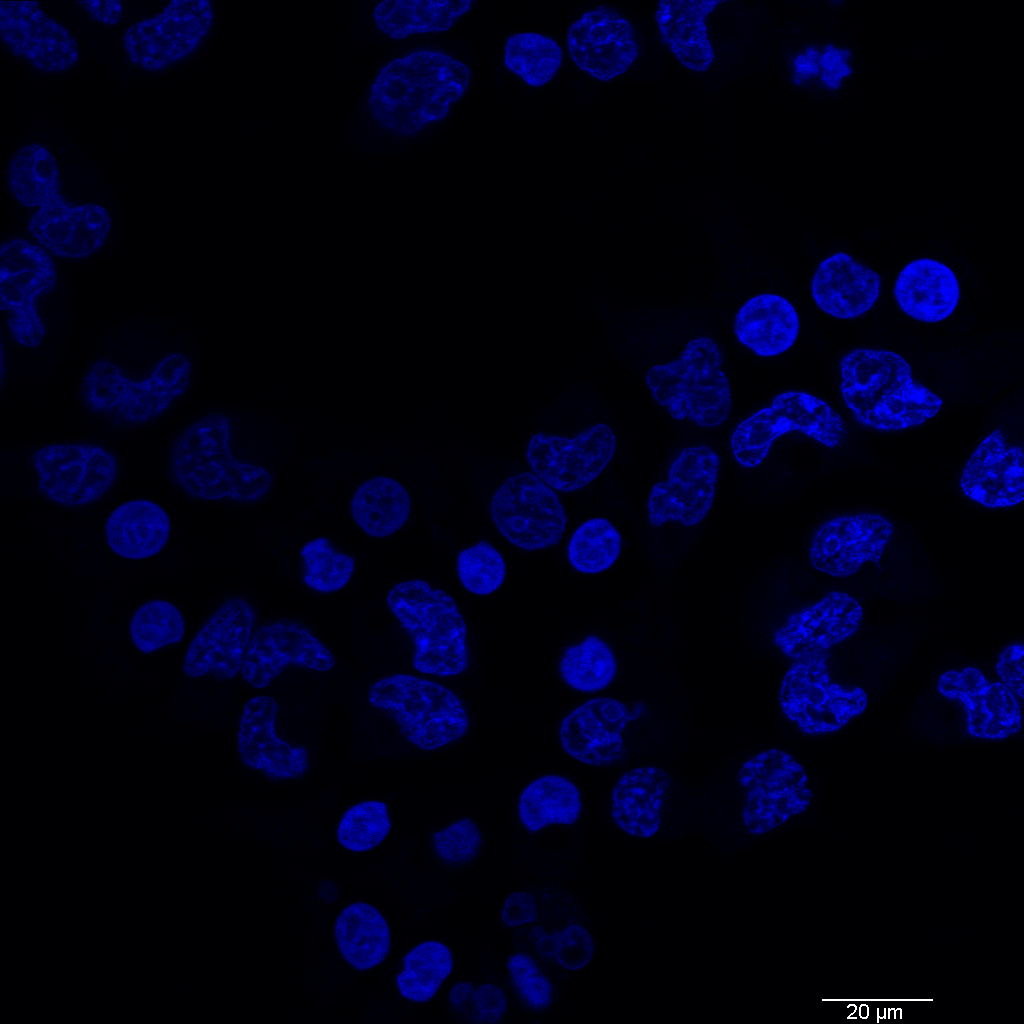

Supplement: Supplementary file 6 — Source data Fig. 2 [file 44318_2025_438_MOESM6_ESM.zip › Figure 2/Figure 2A/rpTx1-Ntermi DAPI.tif]

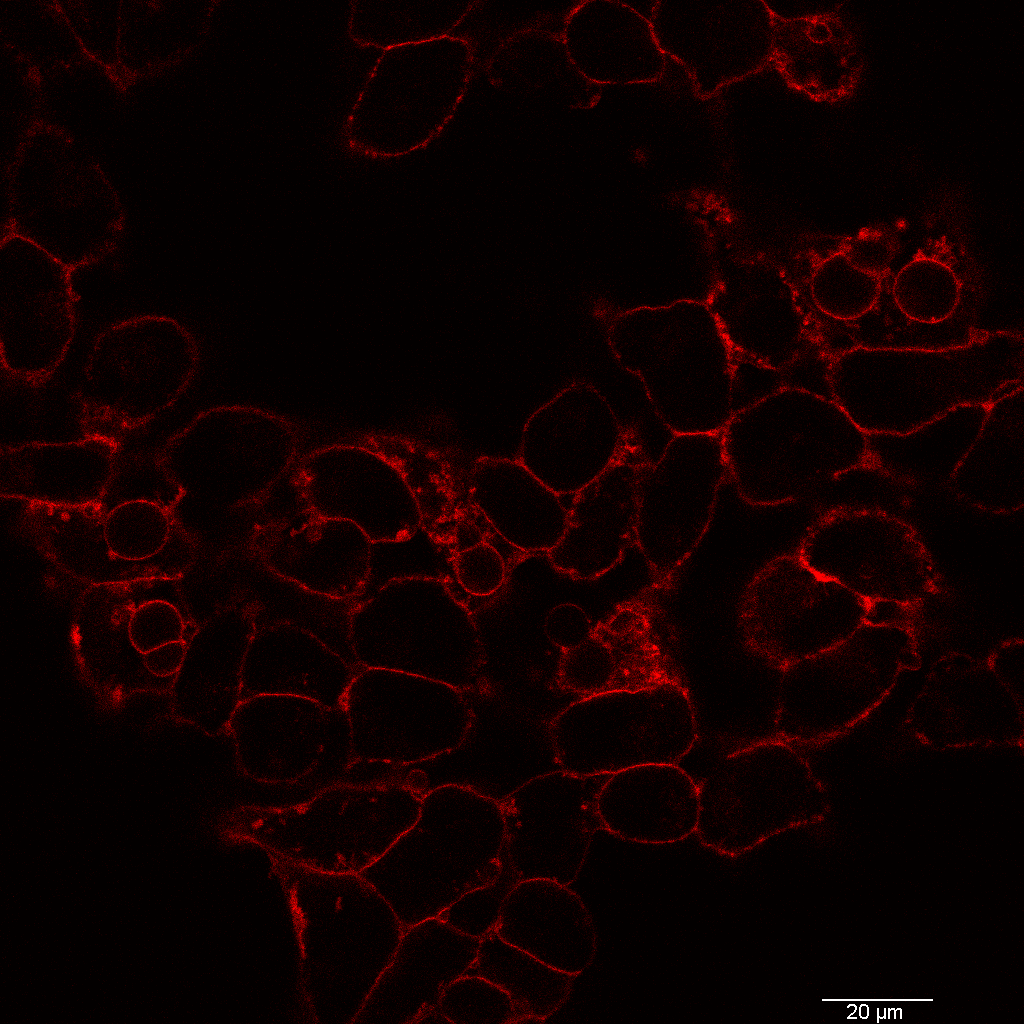

Supplement: Supplementary file 6 — Source data Fig. 2 [file 44318_2025_438_MOESM6_ESM.zip › Figure 2/Figure 2A/rpTx1-Ntermi DiD.tif]

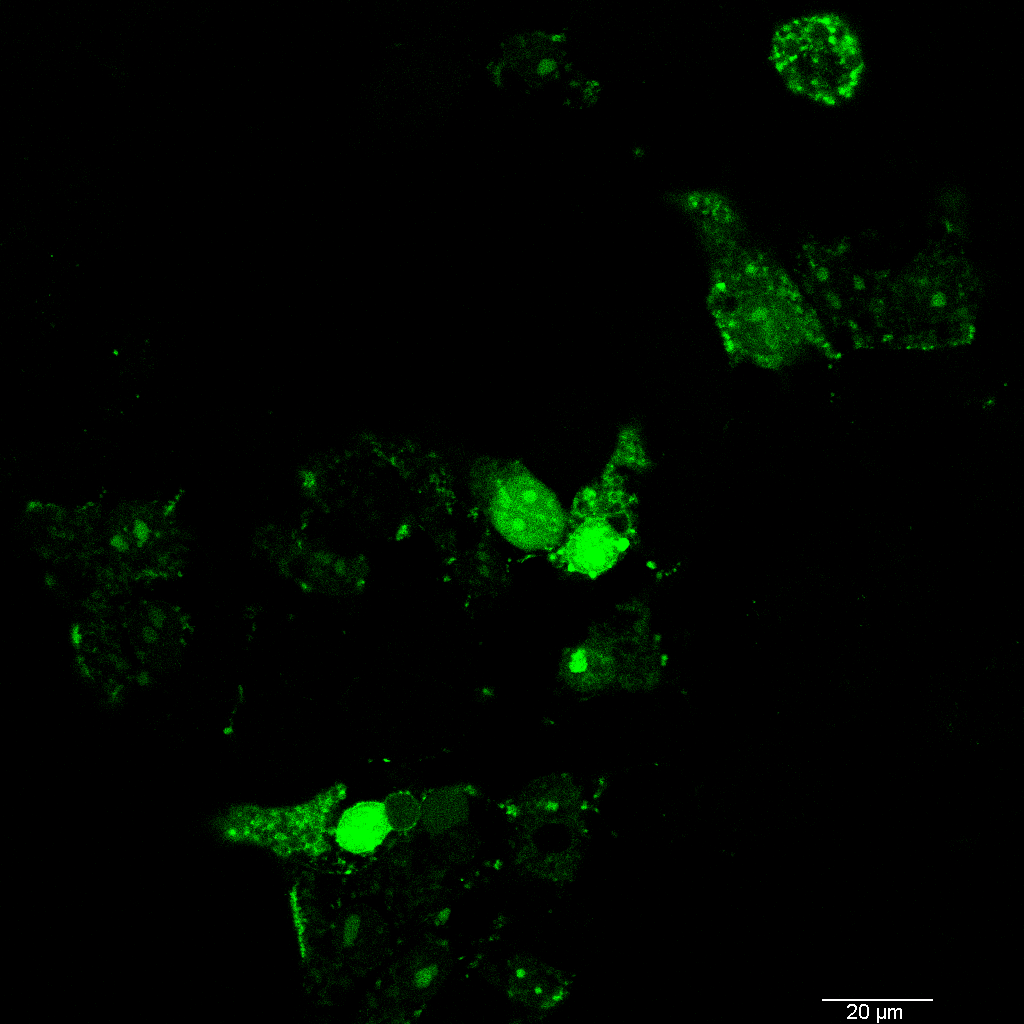

Supplement: Supplementary file 6 — Source data Fig. 2 [file 44318_2025_438_MOESM6_ESM.zip › Figure 2/Figure 2A/rpTx1-Ntermi FITC.tif]

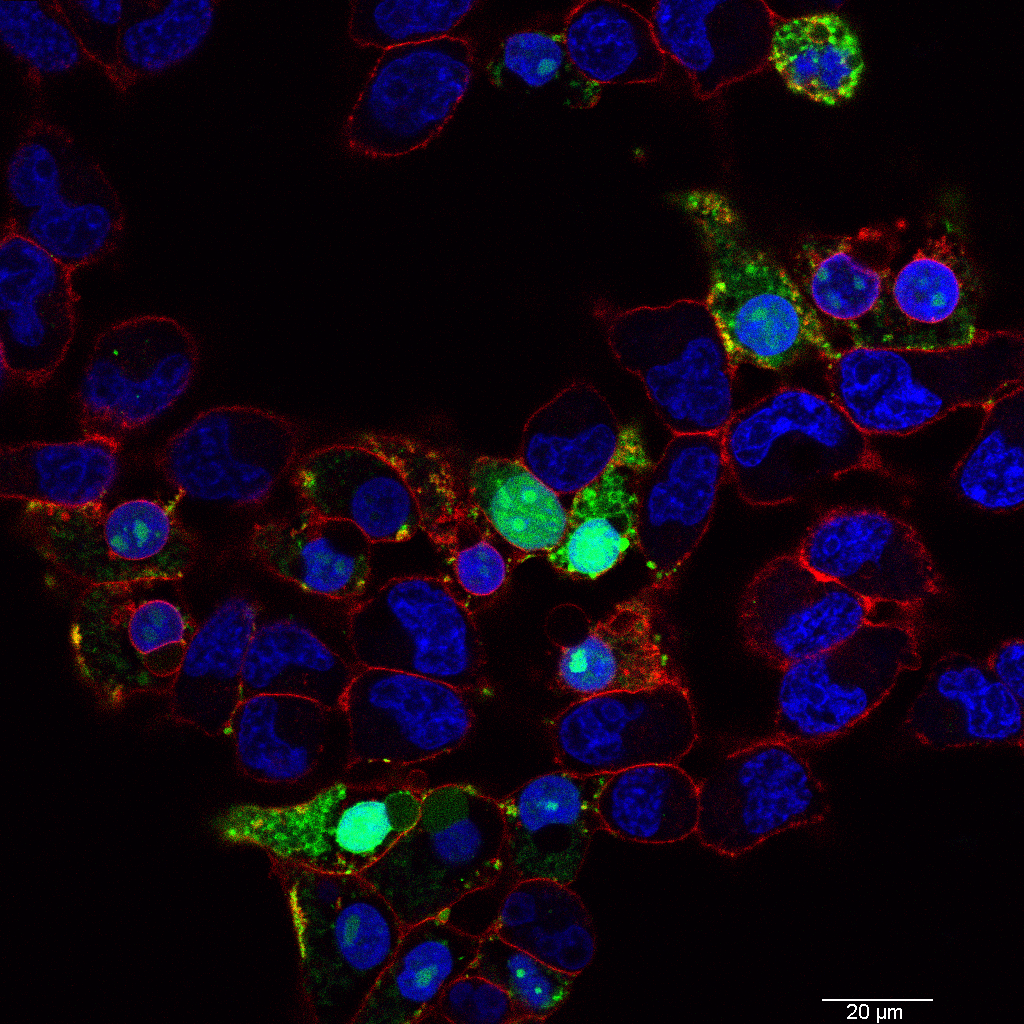

Supplement: Supplementary file 6 — Source data Fig. 2 [file 44318_2025_438_MOESM6_ESM.zip › Figure 2/Figure 2A/rpTx1-Ntermi merge.tif]
