# Supplementary material for: Inherent fast inactivation particle of Nav channels as a new binding site for a neurotoxin
Source: EMBO J. 2025 Apr 22;44(11):3180–209. doi: 10.1038/s44318-025-00438-9 (PMC12130229; doi:10.1038/s44318-025-00438-9)
Supplement: Supplementary file 11 — Expanded View Figures [file 44318_2025_438_MOESM11_ESM.pdf]

Expanded View Figures

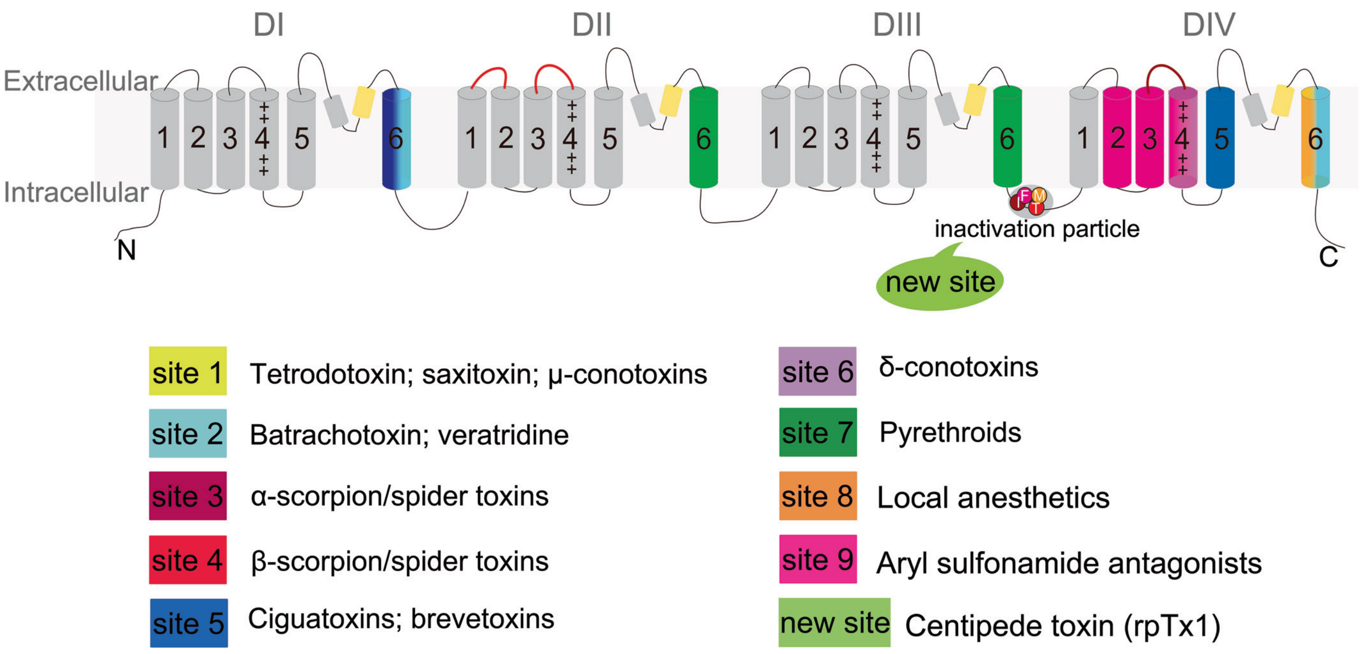

Figure EV1. Neurotoxin/drug receptor sites on Nav channels.

The IFMT motif is a new neurotoxin binding site identified in this study (new site). Adapted from Catterall et al (2007)[21] and Klint et al (2012)[14].

 © The Author(s)

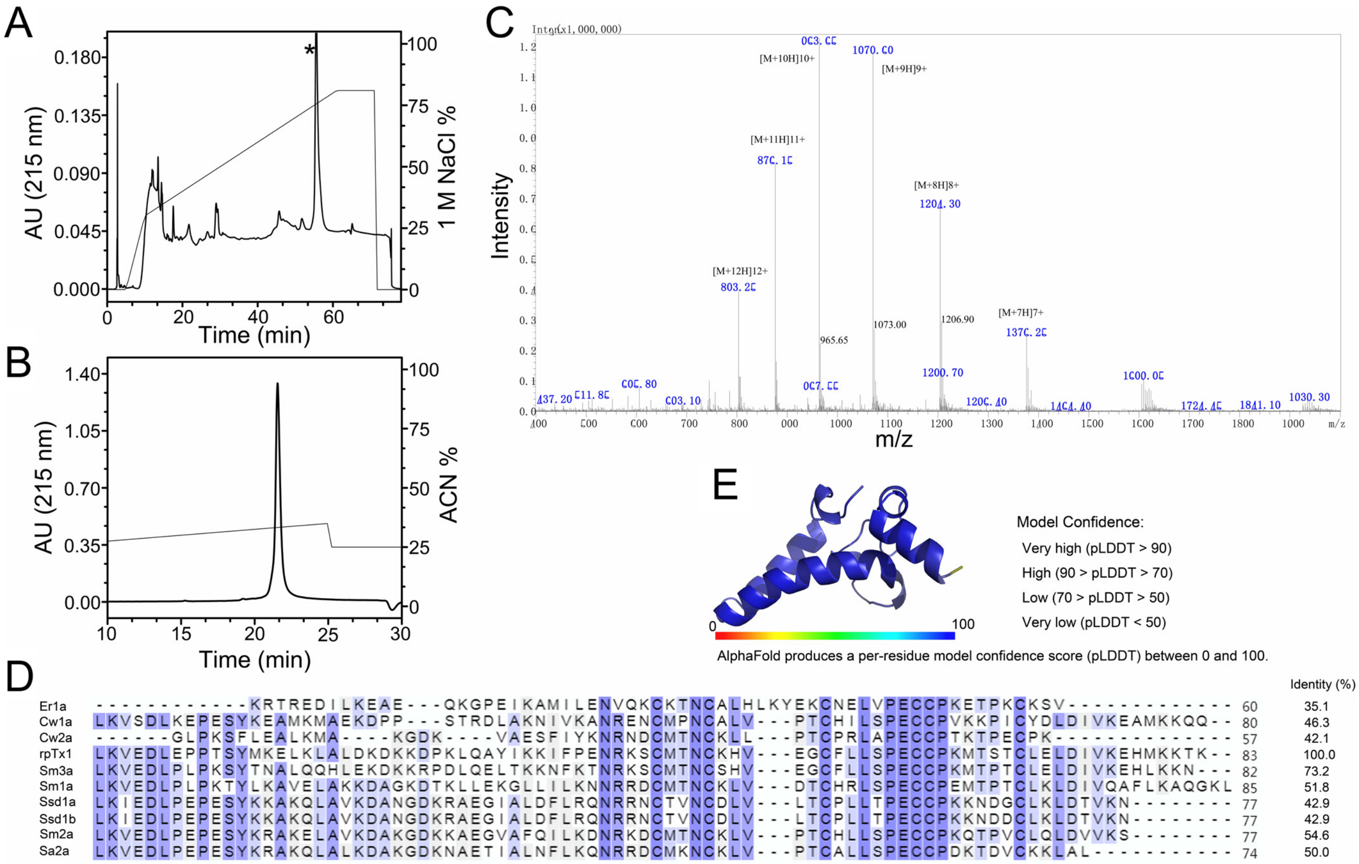

Figure EV2. Purification and characterization of rpTx1.

(**A**) RpTx1 was purified to homogeneity by cation-exchange HPLC. (**B**) Further purification and desalting of the active fraction from (**A**) by RP-HPLC. (**C**) ESI mass spectra of the purified rpTx1. (**D**) Sequence alignment of rpTx1 with several centipede toxins. (**E**) High or very high confidence scores are yielded for the rpTx1 structure predicted by Alphafold2.

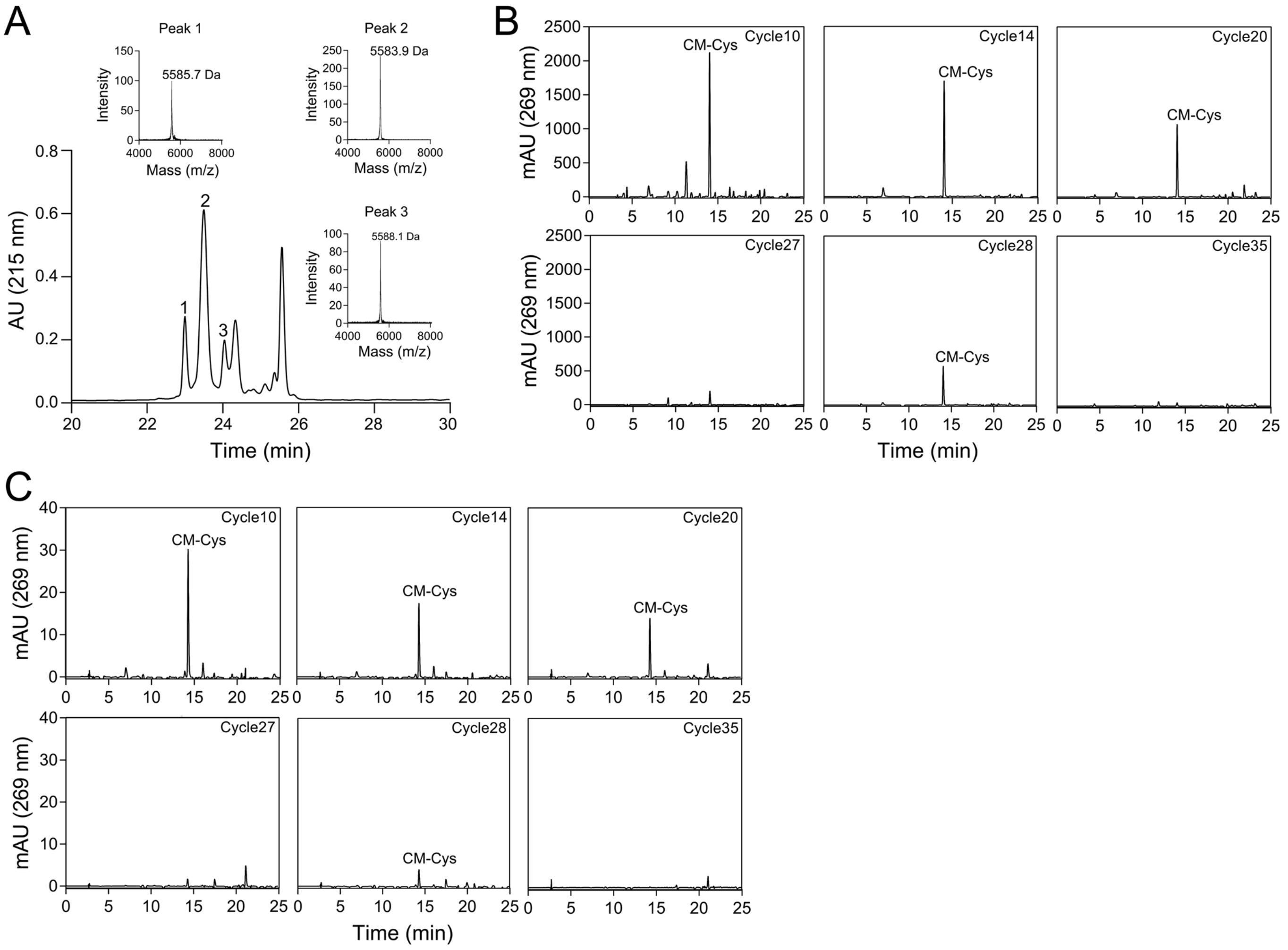

Figure EV3. The determination of disulfide bonds of rpTx1.

(**A**) RP-HPLC chromatogram of the partially reduced products of the truncated rpTx1 (sequence in the section of method), and the MALDI-TOF MS analysis of fractions labeled (insets). These results indicate that the peak 1 is a peptide with one disulfide bond reduced, peak 2 is nonreduced peptide, and peak 3 is a two disulfide bonds reduced peptide. (**B**, **C**) Edman degradation sequencing of the partially reduced peptides after their free sulfydryl groups were alkylated by iodoacetamide. Cysteine residues occur at cycle 10, 14, 20, 27, 28, and 35, respectively, indicating on the upper right of panel. (**B**) The cysteine residue cycles of the alkylated peak 3, a rpTx1 analogue with one disulfide bond, and signals of the alkylated Cys residues (PTH-CM-Cys) are observed in cycle 10, 14, 20 and 28, but not in cycle 27 and 35, indicating that the two disulfide bonds formed by cystine10, 14, 20 and 28 were reduced by TCEP, while the remained disulfide bond Cys27–Cys35 was kept intact. Additionally, we analyzed the decay rates of the four PTH-CM-Cys signals in the Peak 3 Edman degradation sequencing, showing that compared to cycle 10, the signal yield was attenuated by 19% for cycle 14, 50% for cycle 20, and 73% for cycle 28. This is likely the normal signal decay occurring during sequencing. (**C**) Next, we continued to determine the second disulfide bond of rpTx1 by the sequencing of alkylated Peak 1, a rpTx1 analogue with two disulfide bonds. Theoretically, PTH-CM-Cys signals should only appear in two cycles because this peak has only one disulfide bond reduced and alkylated. However, PTH-CM-Cys signals are observed in the chromatogram at the cycle 10, 14, 20, and 28, suggesting that this peak should be a mixture of two fractions, each containing two disulfide bonds, one of which is Cys27–Cys35, and the other one should be different. We attempted to infer the disulfide bond reduced in the two fractions based on the signal intensities of the four PTH-CM-Cys which should correlate with the relative abundance of the two fractions in the mixture and the normal signal decay during sequencing. compared to cycle 10, the signal yield was attenuated by 42% for cycle 14, 54% for cycle 20, and 87% for cycle 28, which shows different pattern from that shown in (**B**). Specifically, compared to Cycle 10, the PTH-CM-Cys signal decays in Cycles 14 and 28 are significantly greater than the normal decay, while the signal decay in Cycle 20 is similar to the normal one. This suggests that the PTH-CM-Cys signals in Cycle 10 and Cycles 14 and 28 originate from two different fractions, with the fraction containing the Cycle 10 PTH-CM-Cys signal being more abundant than the fraction containing the PTH-CM-Cys signals from Cycles 14 and 28, while the PTH-CM-Cys signals in Cycle 10 and Cycle 20 likely originate from the same fraction. Based on these results, it can be inferred that Cys10 and Cys20, as well as Cys14 and Cys28, respectively, form disulfide bonds.

 © The Author(s)

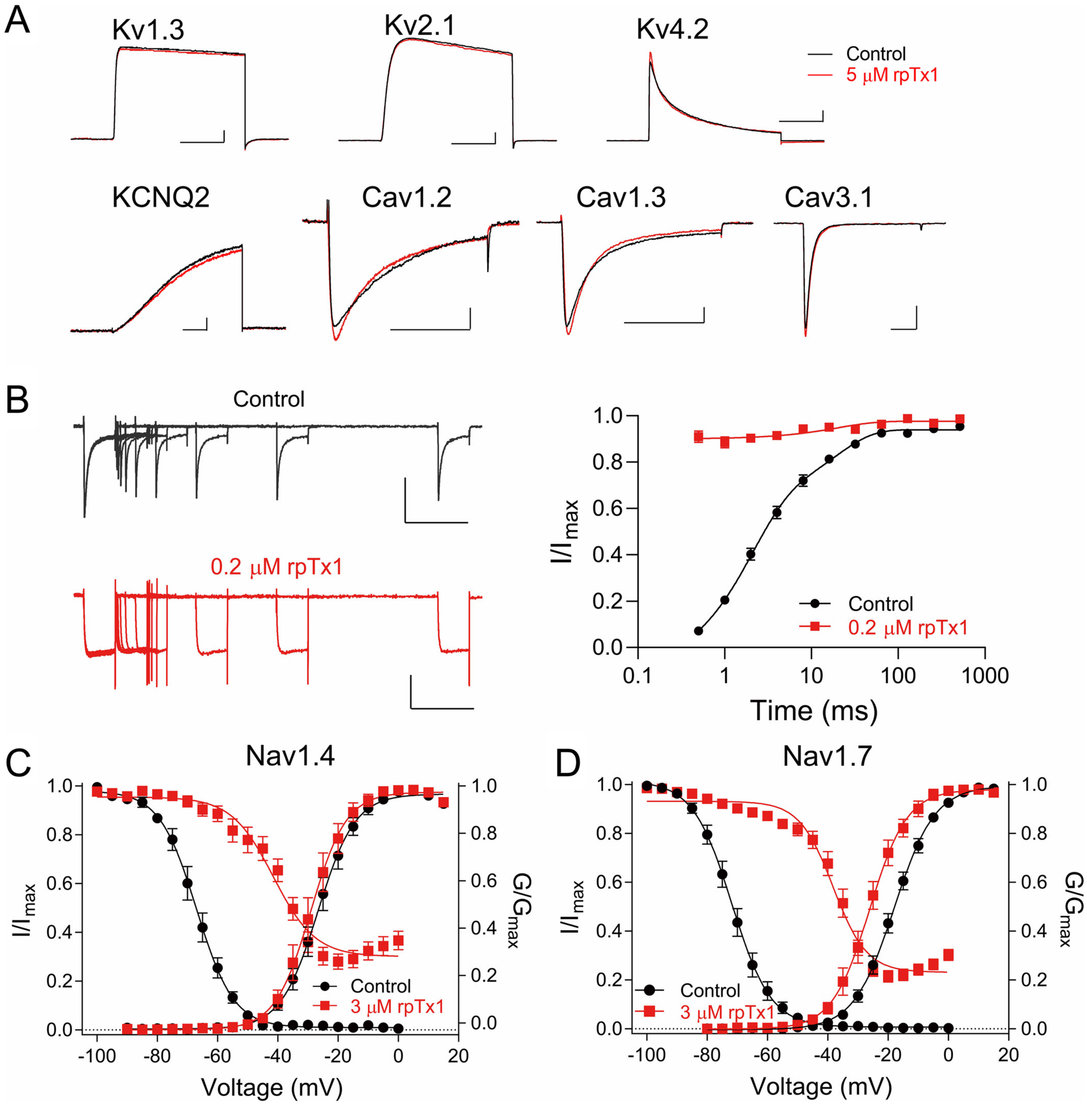

Figure EV4. The effects of rpTx1 on other ion channels, on the fast inactivation recovery of rNav1.8, and on the steady-state activation and inactivation of rNav1.4 and hNav1.7.

(**A**) RpTx1 has no effect on the currents of voltage-gated potassium channels (mKv1.3, rKv2.1, rKv4.2, and hKCNQ2) or voltage-gated calcium channels (mCav1.2, rCav1.3, and rCav3.1) ($n = 3$–5 per group). Scale bar: 100 pA/pF, 100 ms (upper panel), and 20 pA/pF, 50 ms (lower panel). (**B**) Left: representative inactivation recovery current traces of rNav1.8 channels in the absence or presence of 0.2 μM rpTx1. Right: time course of recovery from the fast inactivation of rNav1.8 in the absence ($n = 9$) or presence ($n = 5$) of 0.2 μM rpTx1. Scale bar: 1 nA, 100 ms. (**C**, **D**) The effect of rpTx1 on the steady-state activation (rNav1.4: $n = 9$ for control, $n = 8$ for rpTx1; hNav1.7: $n = 12$ for control, $n = 7$ for rpTx1) and inactivation (rNav1.4: $n = 6$ for control, $n = 8$ for rpTx1; hNav1.7: $n = 14$ for control, $n = 7$ for rpTx1) of rNav1.4 (**B**) and hNav1.7 (**C**). Data are presented as mean ± S.E.M.

© The Author(s)

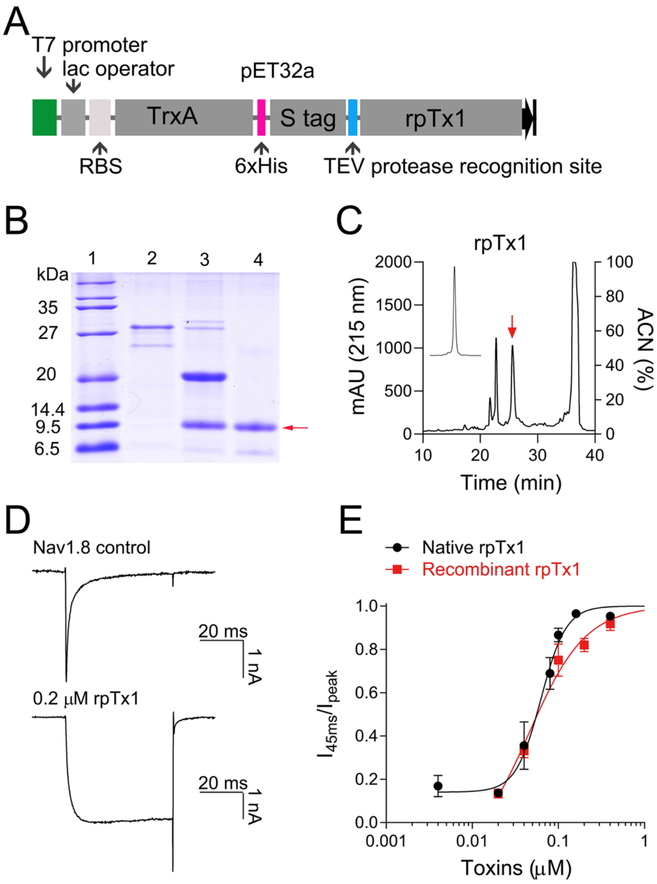

Figure EV5. Recombinant production of rpTx1.

(**A**) Architecture of the pET32a vector used for recombinant expression of rpTx1. The coding region includes a TrxA protein, a 6×His tag, a S-tag, a TEV protease recognition site, and a codon-optimized gene encoding rpTx1. (**B**) Tricine-SDS-PAGE analysis of rpTx1 expressed in BL21 (DE3). Lane 1, molecular weight markers; lane 2, elution fraction with 250 mM imidazole; lane 3, fusion protein after TEV protease cleavage; lane 4, eluted rpTx1 from RP-HPLC. (**C**) RP-HPLC characterization of recombinant rpTx1. The red arrow-labeled peak indicates recombinant rpTx1. Recombinant rpTx1 was further purified to homogeneity by analytical RP-HPLC (inset). (**D**) Representative traces of current traces recorded from ND7/23 cells expressing rNav1.8 in the absence or presence of 0.2 μM recombinant rpTx1 in intracellular solution. (**E**) The concentration–response curves of recombinant rpTx1 and native rpTx1 inhibiting the fast inactivation of rNav1.8 ($n = 3$–10 per concentration). Data are presented as mean ± S.E.M.

 © The Author(s)

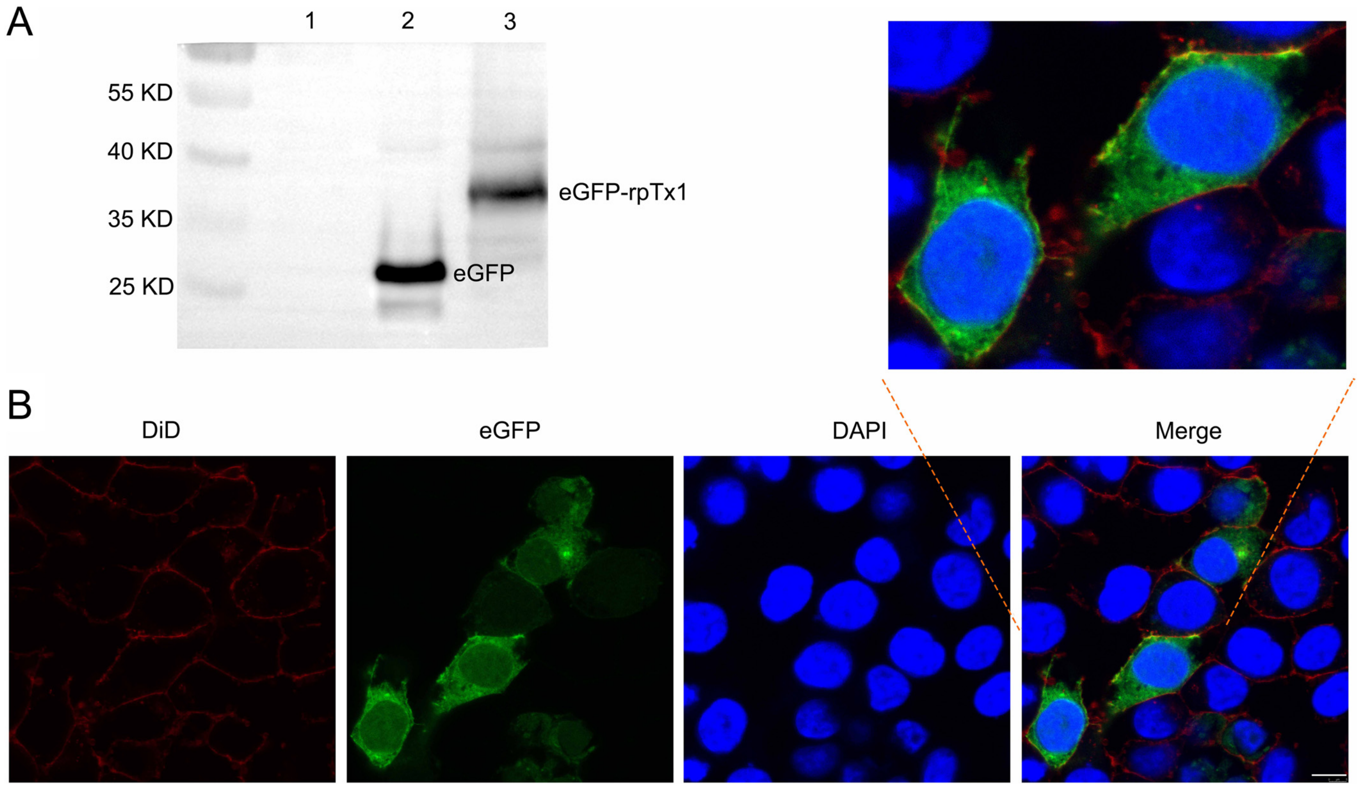

Figure EV6. The eGFP-rpTx1 fusion protein maintained rpTx1 on the intracellular side of cells.

(**A**) Representative western blotting images of recombinant eGFP in cell lysates from HEK293T cells (line 1), HEK293T cells expressing eGFP (line 2), and HEK293T cells expressing eGFP-rpTx1 (line 3), respectively ($n = 3$). (**B**) Confocal images show that eGFP-rpTx1 is primarily distributed within the cytoplasm. DiD and DAPI were used as membranous and nuclei markers, respectively. Scale bar: 10 μm.

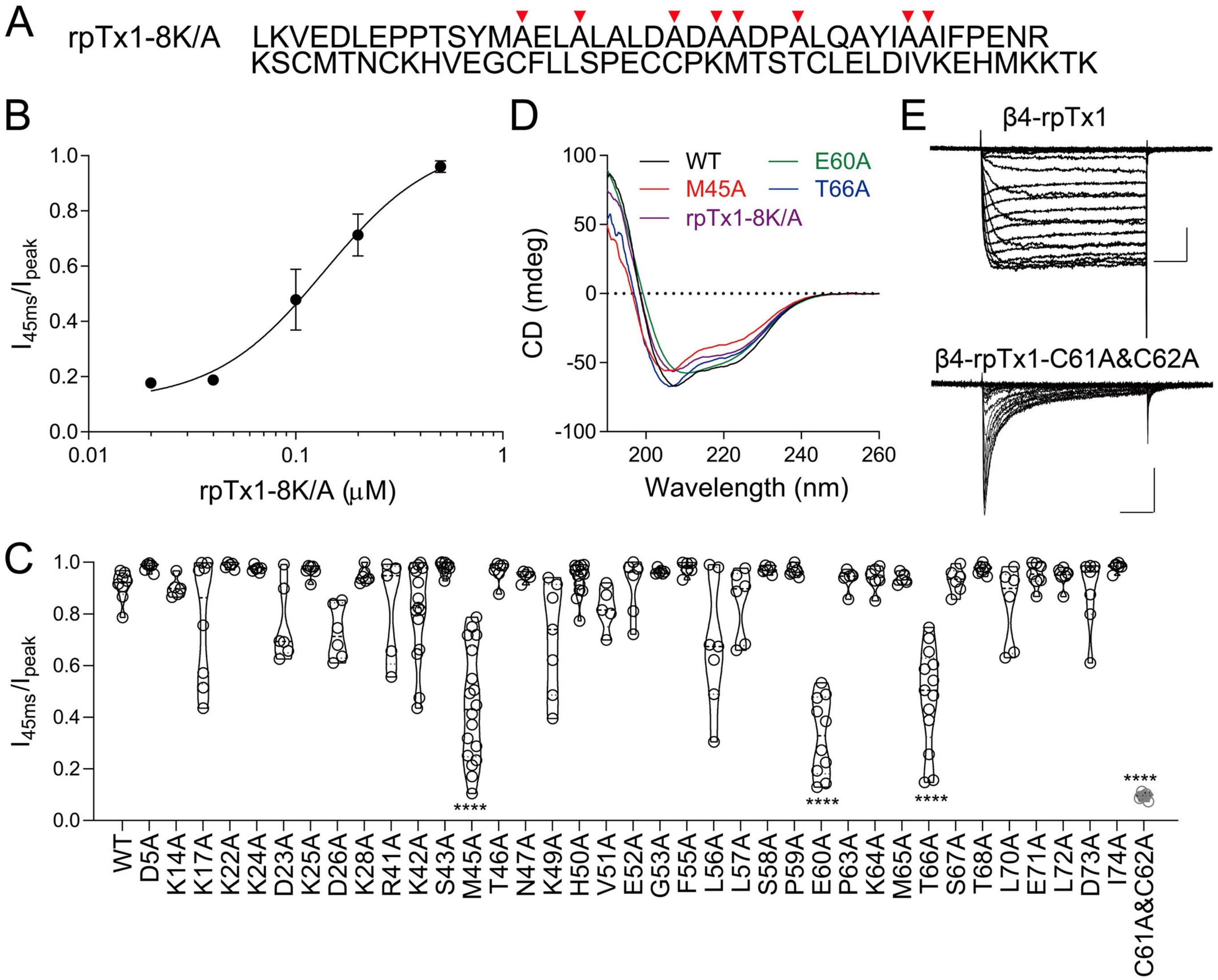

Figure EV7. The effect of rpTx1 mutants on the fast inactivation of rNav1.8.

(**A**) The sequence of rpTx1-8K/A with mutation sites labeled by red arrows. (**B**) Concentration-dependent curves show the effect of the intracellular application of rpTx1-8K/A on rNav1.8 ($n = 4$–11 per concentration). (**C**) Potency of 0.1 μM WT rpTx1 and mutants measured on rNav1.8. Note that M45A, E60A, T66A and C61A&C62A mutations remarkably reduced toxin's availability on the channel (one-way ANOVA with post hoc analysis using Dunnett's multiple comparisons test, $n = 5$–18). (**D**) CD spectra of WT and rpTx1 mutants. (**E**) Representative traces of current families were recorded from ND7/23 cells expressing rNav1.8 co-expressed with 3.5 μg β4-rpTx1 ($n = 5$) or β4-rpTx1-C61A&C62A ($n = 6$). Scale bar: 0.5 nA, 10 ms. Data are presented as mean ± S.E.M. ****$p < 0.0001$.

 © The Author(s)

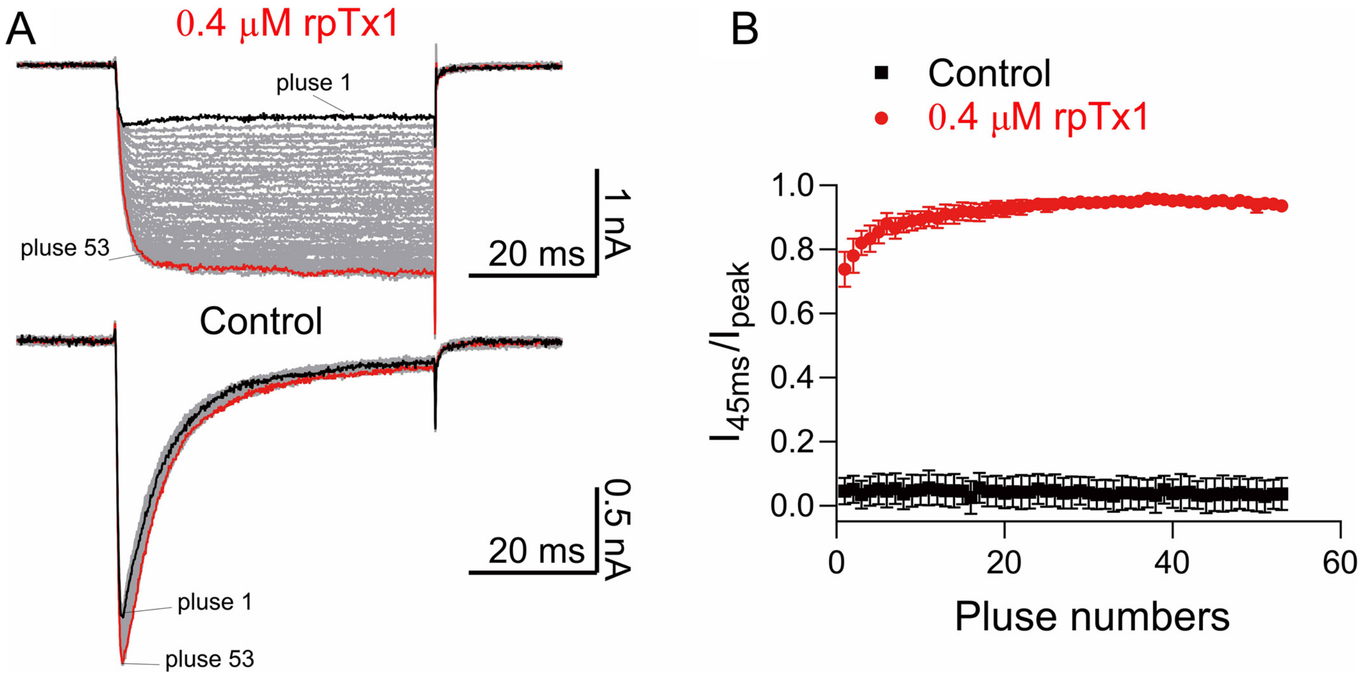

Figure EV8. RpTx1 rapidly inhibits the fast inactivation of rNav1.8.

(**A**) Representative current traces of repetitive test pulse at 20 mV from the holding potential at −90 mV at 0.2 Hz frequency in the presence (upper) or absence (lower) of 0.4 μM rpTx1 in pipette. Patch-clamp recordings were performed immediately after establishing a whole-cell configuration on ND7/23 cells expressing rNav1.8. (**B**) Time-course of inhibition of rNav1.8's fast inactivation by 0.4 μM rpTx1 ($n = 6$ for rpTx1, $n = 5$ for control).

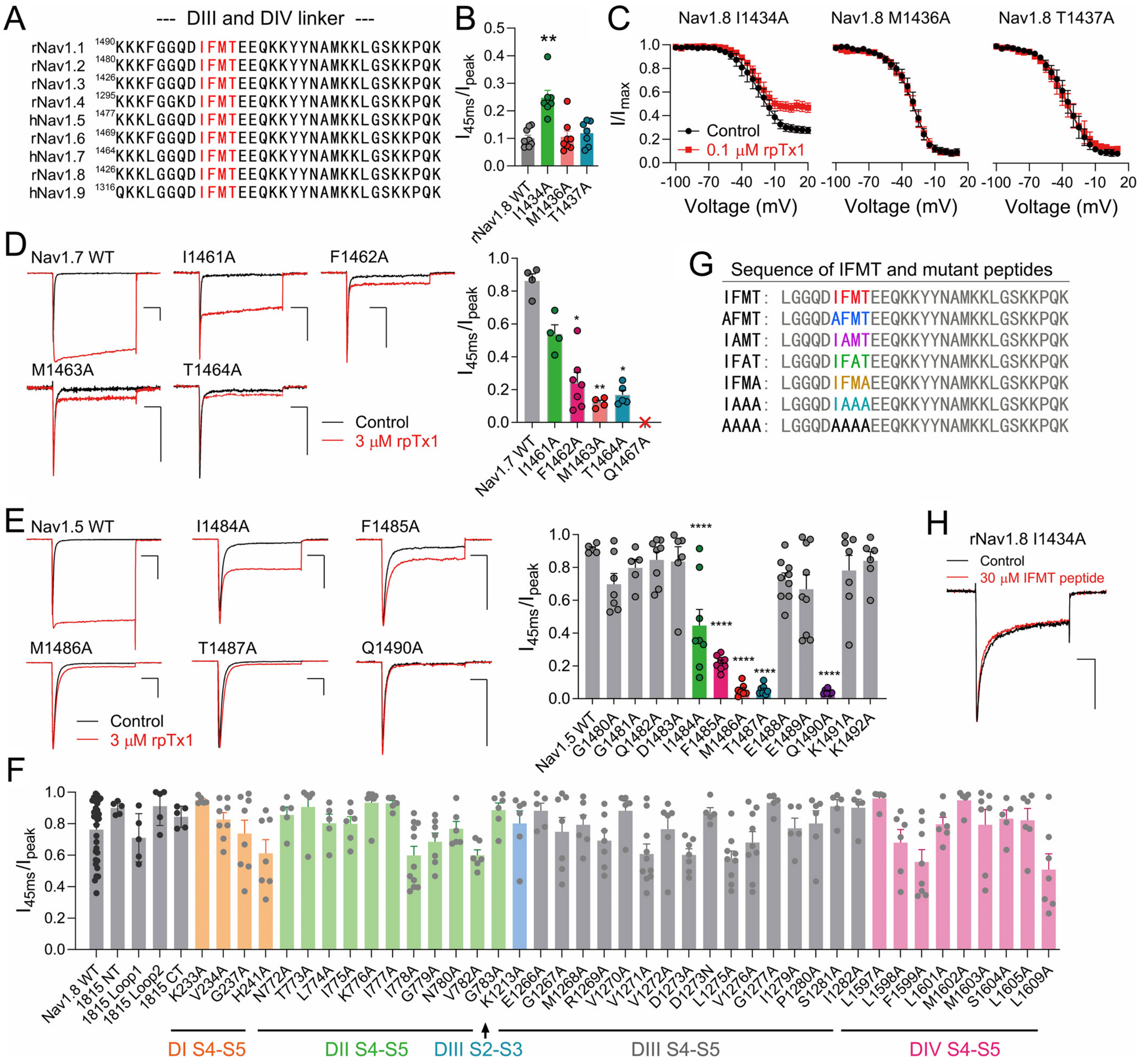

Figure EV9. The IFMT motif of Nav channels is the key region for the action of rpTx1.

(**A**) Sequence alignments corresponding to the fast inactivation gate region (DIII–DIV linker) of Nav channel subtypes. The red highlighted sequences show the IFMT motif. (**B**) The scatter dot plot shows the effect of rNav1.8 mutants I1434A ($n = 7$), M1436A ($n = 8$), and T1437A ($n = 7$) on the fast inactivation of the channel (Non-parametric test using Dunn's multiple comparisons test). (**C**) The effect of rpTx1 in the pipette on the steady-state inactivation of rNav1.8 mutants I1434A ($n = 9$ for control, $n = 8$ for rpTx1), M1436A ($n = 5$ for control, $n = 6$ for rpTx1) and T1437A ($n = 5$ for control, $n = 6$ for rpTx1). (**D**) Representative current traces from WT and mutant hNav1.7 channels in the absence or presence of 3 μM rpTx1 in pipette. Scatter dot plot show the effect of 3 μM rpTx1 on the persistent currents ($I_{45\ ms}/I_{peak}$) of WT and mutant hNav1.7 channels (Non-parametric test using Dunn's multiple comparisons test, $n = 3$–7). Scale bar: 1 nA, 10 ms. (**E**) Representative current traces from WT and mutant hNav1.5 channels in the absence or presence of 3 μM rpTx1in pipette. Scatter dot plot show the effect of 3 μM rpTx1 on the persistent currents ($I_{45\ ms}/I_{peak}$) of WT and mutant hNav1.5 channels (one-way ANOVA with post hoc analysis using Dunnett's multiple comparisons test, $n = 5$–9). Scale bar: 2 nA, 10 ms. (**F**) Scatter dot plot show the effect of 0.1 μM rpTx1in pipette on the persistent currents ($I_{45\ ms}/I_{peak}$) of WT and mutant rNav1.8 channels (Non-parametric test using Dunn's multiple comparisons test, $n = 5$–32). (**G**) Sequences of the IFMT peptide and its 6 mutants. The mutated amino acid residues are highlighted in colors. (**H**) Representative current traces showing the effect of 30 μM IFMT peptide on rNav1.8 I1434A upon intracellular application ($n = 4$). Scale bar: 1 nA, 10 ms. Data are presented as mean ± S.E.M. **$p < 0.01$, ***$p < 0.001$, ****$p < 0.0001$.

 © The Author(s)

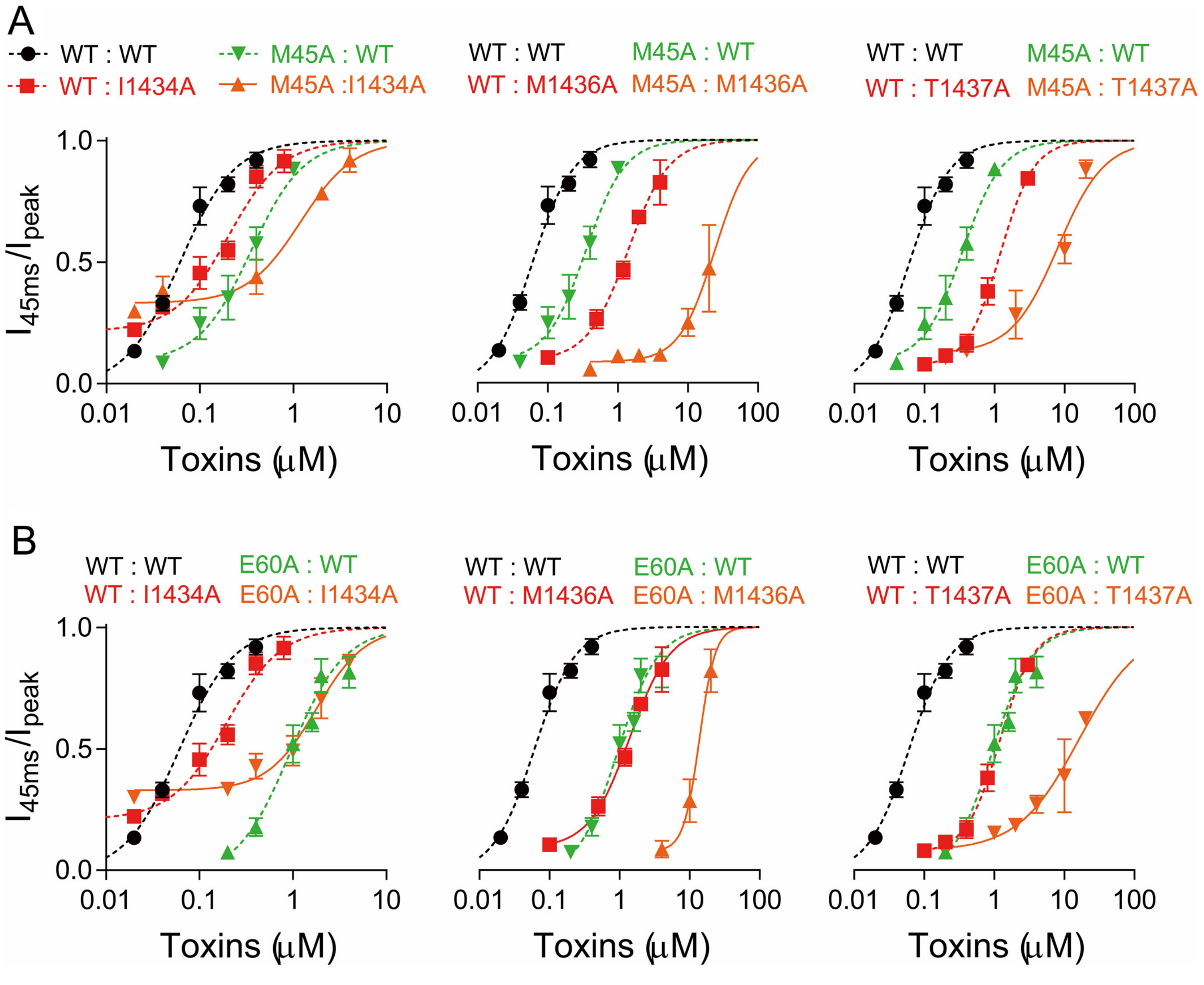

Figure EV10. Mutant cycle analysis for pairwise coupling between rpTx1 and rNav1.8.

Concentration–response curves for determining the interaction Ln(Ω) values between the rpTx1 M45 and rNav1.8 I1434 or M1436 or T1437 pair (**A**), the rpTx1 E60 and rNav1.8 I1434 or M1436 or T1437 pair (**B**). $n = 3$–17 per concentration. Data are presented as mean ± S.E.M.

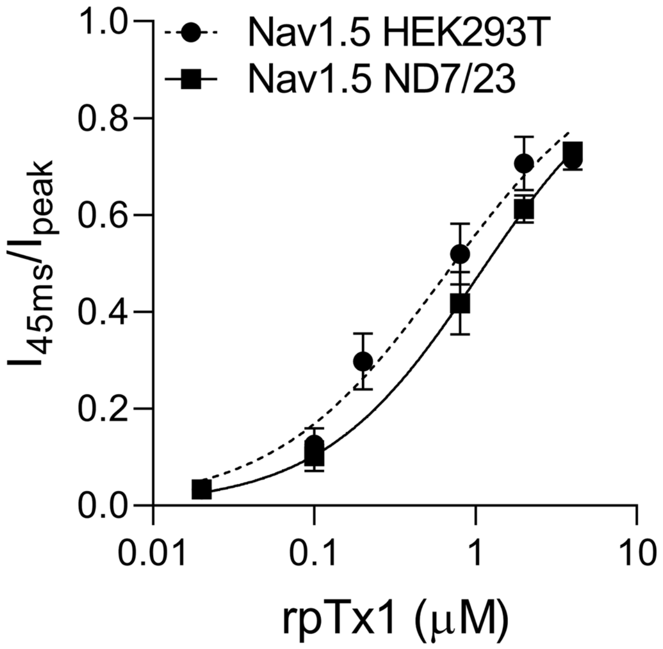

Figure EV11. The effect of rpTx1 on the activity of hNav1.5 expressed in different cell types.

The concentration-dependent curves show the effect of rpTx1 in pipette on hNav1.5 expressed in either HEK293T or ND7/23 cells ($n = 3$–8 per concentration). Data are presented as mean ± S.E.M.

 © The Author(s)
